# New Bioactive Sesquiterpeniods From the Plant Endophytic Fungus *Pestalotiopsis theae*

**DOI:** 10.3389/fmicb.2021.641504

**Published:** 2021-03-31

**Authors:** Gaoran Liu, Ruiyun Huo, Yanan Zhai, Ling Liu

**Affiliations:** ^1^State Key Laboratory of Mycology, Institute of Microbiology, Chinese Academy of Sciences, Beijing, China; ^2^College of Life Sciences, University of Chinese Academy of Sciences, Beijing, China

**Keywords:** secondary metabolites, endophytic fungi, sesquiterpenoids, electronic circular dichroism, bioactivity, *Pestalotiopsis theae*

## Abstract

Three new secondary metabolites pestalothenins A–C (**1**–**3**), including two new humulane-derived sesquiterpeniods (**1** and **2**) and one new caryophyllene-derived sesquiterpeniod (**3**), together with five known compounds (**4**–**8**) were isolated from the crude extract of the plant endophytic fungus *Pestalotiopsis theae* (N635). Their structures were elucidated by the extensive analyses of HRESIMS and NMR spectroscopic data. The absolute configurations of **1**–**3** were determined by comparison of experimental and calculated electronic circular dichroism (ECD) spectra. The cytotoxic effects of these compounds were evaluated *in vitro*. Compound **6** showed moderate cytotoxicity against T24 and MCF7 cell lines. In addition, compounds **1**–**8** were also evaluated for antibacterial activity.

## Introduction

Sesquiterpenoids are a group of naturally occurring 15-carbon isoprenoids, showing diverse structural features and interesting bioactivities (Fraga, 2011, 2013). Humulane-type sesquiterpenoids represent an uncommon type of compound possessing a characteristic 11-membered ring in the molecule, which display a wide range of bioactivities including antibacterial, antifungal, cytotoxic, and immunosuppressive activities (Pulici et al., 1996a; Toyota et al., 2004; Luo et al., 2006; Liao et al., 2013; Chen et al., 2014; Wang et al., 2017; Kapustina et al., 2020). Caryophyllene-derived sesquiterpenoids are a group of structurally unique compounds characterized by the presence of a bicyclo[2.7.0]undecane skeleton, which are believed to be derived from humulane (Ayer and Browne, 1981; Daniewsk et al., 1981). Some caryophyllene derivatives exhibited cytotoxic, immunosuppressive, analgesic, and antimicrobial activities (Collado et al., 1994; Pulici et al., 1996b; Fidyt et al., 2016). To date, different humulane-type and caryophyllene-derived sesquiterpenoids have been found in plants (Ghalib et al., 2012; Liao et al., 2013), liverworts (Bardón et al., 1999; Toyota et al., 2004), and fungi (Chen et al., 2014; Guo et al., 2020b). In recent years, these two types of sesquiterpenoids have attracted increasing attention and have become the challenging targets of total synthesis (Takao et al., 2008, 2009).

**FIGURE 1:**
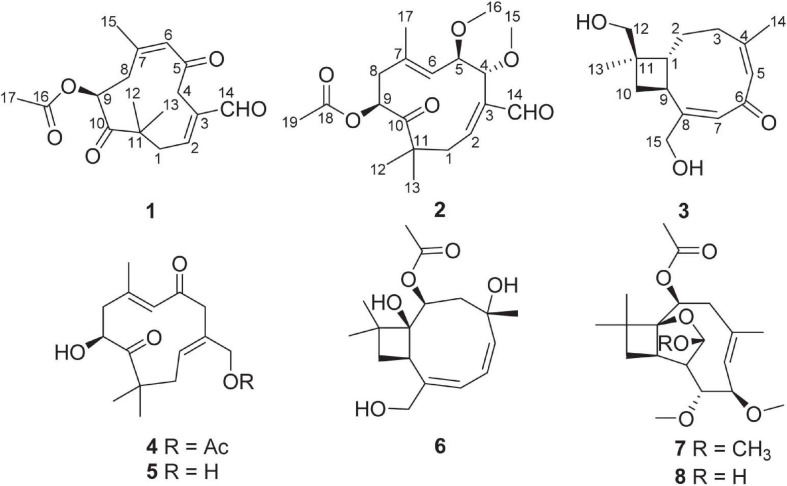
Chemical structures of compounds **1–8**.

Fungi have contributed significantly to drug discovery as major sources of lead compounds with inspiring novel structures (Jiang et al., 2018; Wu et al., 2018; Han et al., 2019; Liu et al., 2019; Luo et al., 2019). Plant endophytic fungi, the major group of special environmental fungi inhabiting living plants without any negative effects, have been proven to be a rich source of structurally unique and bioactive secondary metabolites (Petrini et al., 1992; Strobel, 2003; Strobel et al., 2004). The widely distributed endophytic fungi, *Pestalotiopsis* spp., has attracted much attention owing to the discovery of structurally diverse and biologically active secondary metabolites (Liu et al., 2009, 2010). Some caryophyllene type sesquiterpenoids, such as pestaloporinates A–G and pestaloporonins A–C, have also been reported from *Pestalotiopsis* (Hwang et al., 2015; Liu et al., 2016b). In our search for new bioactive secondary metabolites from this fungal genus, a strain of *P*. *theae* (N635), isolated from the branches of the tea plant *Camellia sinensis* (Theaceae) in the suburb of Hangzhou, P. R. China, was grown in different solid-substrate fermentation. Chemical studies of the resulting crude extracts had afforded two cytotoxic spiroketals and their putative biosynthetic precursors (Liu et al., 2016a), nine cytotoxic and antioxidant polyketides (Guo et al., 2020a), and five cytotoxic caryophyllene-derived sesquiterpenoids with 4/6/5 ring system (Guo et al., 2020b). Further chemical investigations of these fractions led to the isolation of two new humulane-derived sesquiterpeniods, pestalothenins A (**1**) and B (**2**), one new caryophyllene-derived sesquiterpeniod pestalothenin C (**3**), together with five known compounds, 14-acetylhumulane (**4**) (Liu et al., 2016b), 9,15-Dihydroxy-2,6-humuladiene-5,10-dione (**5**) (Pulici et al., 1996a), punctaporonin H (**6**) (Wu et al., 2014), pestalotiopsin E (**7**) (Xiao et al., 2017), and pestalotiopsin C (**8**) (Pulici et al., 1997; Figure 1). Here we report the isolation, structure elucidation and biological activities of these compounds.

## Materials and Methods

### General Experimental Procedures

IR data were recorded using a Nicolet IS5 FT-IR spectrophotometer. NMR spectra were recorded on a Bruker Avance spectrometer operating at 400, 500, or 600 MHz with tetramethylsilane as an internal standard. HRESIMS data was obtained using an Agilent Accurate-Mass-Q-TOF LC/MS 6520 instrument. Optical rotation was measured by an Anton Paar MCP 200 Automatic Polarimeter. Silica gel (200–300 mesh, Qingdao Ocean Chemical Co., Ltd., China) and Sephadex LH-20 (Amersham Biosciences) were used for column chromatography (CC). HPLC analysis was performed with the Waters 2489 HPLC system using an octadecylsilyl (ODS) column (Pack, ReproSil-Pur Basic, 250 × 4.6 mm, 5 μm) with a flow rate of 1.0 mL/min. HPLC separation was performed on an Agilent HPLC instrument equipped with a variable-wavelength UV detector using an ODS column (C18, 250 × 9.4 mm, 5 μm) with a flow rate of 2.0 mL/min.

### Fungal Material

The fungus *P. theae* has been previously described (Guo et al., 2020a).

### Fermentation, Extraction, and Isolation

The plant endophytic fungus *P. theae* was grown on PDA at 25°C for 10 days, then several pieces of agar plugs (about 0.5 × 0.5 × 0.5 cm^3^) were inoculated into 250 mL Erlenmeyer flasks containing 50 mL of media (0.4% glucose, 1% malt extract, and 0.4% yeast extract) at room temperature on an orbital shaker at 170 rpm for 5 days to produce the seed culture. Finally, 5.0 mL of the spore inoculums obtained from liquid phase cultivation was added to Erlenmeyer flasks (500 mL) containing 80 g of rice and 120 mL of distilled H_2_O and incubated at 25°C for 40 days. The fermented rice material was extracted repeatedly with EtOAc (3 × 4.0 L), and the organic solvent was evaporated to dryness to afford the crude extract (15 g), which was fractionated by silica gel vacuum liquid chromatography (VLC), eluted with a gradient of petroleum ether/EtOAc and EtOAc/MeOH to generate 10 fractions (V1–V10). The fraction V3 (950 mg) eluted with 75% petroleum ether/EtOAc was fractionated on a Sephadex LH-20 column chromatography (CC) eluting with CH_2_Cl_2_:MeOH (1:1) to furnish five subfractions (V3-S1 to V3-S5), and the subfractions V3-S3 (100 mg) was further purified by RP-HPLC (Agilent Eclipse XDB-C18 250 × 9.4 mm, 5 μm column) to provide **1** (*t*_*R*_ 13.8 min, 1.5 mg, 60% MeOH/H_2_O for 15 min, 2.0 mL/min), **2** (*t*_*R*_ 17.8 min, 2.5 mg, 64% MeOH/H_2_O for 34 min, 2.0 mL/min), **7** (*t*_*R*_ 33.2 min, 1.5 mg, 64–84% MeOH/H_2_O for 40 min, 2.0 mL/min) and **8** (*t*_*R*_ 17.2 min, 4.2 mg, 39% CH_3_CN/H_2_O for 25 min, 2.0 mL/min). The fraction V6 (860 mg) eluted with 40% petroleum ether/EtOAc was separated by normal pressure silica gel CC using petroleum ether/EtOAc gradient elution to generate six subfractions (V6-S1 to V6-S6). The subfraction V6-S4 (96 mg) was further purified by RP-HPLC (40% MeOH/H_2_O for 16 min, followed by 58% MeOH/H_2_O for 14 min, 2.0 mL/min) to afford **5** (*t*_*R*_ 16.6 min, 7.5 mg) and **3** (*t*_*R*_ 28.6 min, 13.5 mg). The fraction V9 (780 mg) eluted with 90% petroleum ether/EtOAc was subjected to ODS C-18 CC eluting with MeOH/H_2_O to get five subfractions (V9-S1 to V9-S5), and further the subfraction V9-S5 purified by RP-HPLC to provide **4** (*t*_*R*_ 12.3 min, 5.2 mg, 45–60% MeOH/H_2_O for 30 min, 2.0 mL/min). The fraction V10 (380 mg) eluted with 100% EtOAc was subjected to Sephadex LH-20 CC eluting with MeOH to generate four subfractions (V10-S1 to V10-S4), and further the subfraction V10-S3 was purified by HPLC to provide **6** (*t*_*R*_ 31.9 min, 10.0 mg, 43% MeOH/H_2_O for 35 min, 2.0 mL/min).

*Pestalothenin A (****1***): yellow power; [α]^25^_*D*_ –22.0 (*c* 0.1 MeOH); UV (MeOH) λ_*max*_ (log ε) 237 (2.31) nm; CD (*c* 5.0 × 10^–3^ M, MeOH) λ_*max*_ (Δε) 301 (+ 3.1), 259 (+ 6.9), 226 (–10.9) nm; IR (neat) ν_*max*_ 3,410, 3,352, 2,927, 2,932, 2,880, 2,742, 1,737, 1,712, 1,682, 1,633, 1,248, 1,031 cm^–1^; ^1^H and ^13^C NMR data (see Table 1); HRESIMS *m/z* 329.1373 [M + Na]^+^ (calcd for C_1__7_H_2__2_O_5_Na, 329.1365).

**TABLE 1:** ^1^H and ^13^C NMR data of **1** and **2** in CDCl_3_.

Position	1^*a*^		2^*b*^	
	δ_*H*_ (*J* in Hz)	δ_*C*_, type	δ_*H*_ (*J* in Hz)	δ_*C*_, type
1	2.77, dd (16.0, 12.0)	40.6,CH_2_	3.38, dd (16.8, 12.0)	37.8, CH_2_
	2.16, dd (16.0, 3.0)		1.98, dd (16.8, 3.0)	
2	6.88, dq (12.0, 3.0)	154.2, CH	6.65, dd (12.0, 3.0)	152.3, CH
3		137.2, C		139.3, C
4	3.83, d (15.2)	39.4, CH_2_	4.71, d (3.0)	79.2, CH
	2.85, dt (15.2, 2.0)			
5		203.3, C	4.14, dd (8.4, 3.0)	78.9, CH
6	5.67, s	128.2, CH	4.73, d (8.4)	128.5, CH
7		149.1, C		134.0, C
8	3.65, dd (15.2, 4.5)	31.6, CH_2_	2.85, t (11.5)	40.9, CH_2_
	2.37, dd (15.2, 4.5)		2.13, dd (11.5, 3.0)	
9	5.44, t (4.5)	72.1, CH	5.57, dd (11.5, 3.0)	69.3, CH
10		209.5, C		208.3, C
11		47.3, C		47.5, C
12	1.47, s	26.4, CH_3_	1.36, s	26.4, CH_3_
13	1.32, s	25.6, CH_3_	1.35, s	22.1, CH_3_
14	9.47, s	193.2, CH	9.37, s	194.0, CH
15	1.91, s	25.8, CH_3_	3.26, s	57.2, CH_3_
16		170.5, C	3.24, s	56.7, CH_3_
17	2.07, s	20.7, CH_3_	1.86, s	18.2, CH_3_
18				170.0, C
19			2.06, s	21.0, CH_3_

*^*a*1^H (500 MHz) and ^13^C (125 MHz) NMR data in CDCl_3_.*

*^*b*1^H (600 MHz) and ^13^C (150 MHz) NMR data in CDCl_3_.*

*Pestalothenin B (****2***): colorless oil; [α]^25^_*D*_ –40.0 (*c* 0.07 MeOH); UV (MeOH) λ_*max*_ (log ε) 204 (1.48) nm; CD (*c* 5.0 × 10^–3^ M, MeOH) λ_*max*_ (Δε) 329 (+ 0.7), 228 (–2.7) nm; IR (neat) ν_*max*_ 2,977, 2,935, 2,827, 1,744, 1,720, 1,694, 1,635, 1,230, 1,094 cm^–1^; ^1^H and ^13^C NMR data (see Table 1); HRESIMS *m/z* 375.1882 [M + Na]^+^ (calcd for C_1__9_H_2__8_O_6_Na, 375.1886).

*Pestalothenin C (****3***): colorless oil; [α]^25^_*D*_ –124.0 (*c* 0.1 MeOH); UV (MeOH) λ_*max*_ (log ε) 205 (2.24), 261 (3.72) nm; CD (*c* 3.3 × 10^–3^ M, MeOH) λ_*max*_ (Δε) 336 (+ 3.7), 267 (–9.0), 209 (+ 6.2) nm; ^1^H and ^13^C NMR data (see Table 2); HRESIMS *m/z* 251.1665 [M + H]^+^ (calcd for C_1__5_H_2__3_O_3_, 251.1642).

**TABLE 2:** ^1^H (400 MHz) and ^13^C NMR (100 MHz) data for **3** in methanol-*d*_4._

Position	δ_*H*_ (*J* in Hz)	δ_*C*_, type
1	2.45, dt (11.4, 7.8)	43.0, CH
2	1.71, m	22.8, CH_2_
	1.56, m	
3	2.86, t (11.4)	29.4, CH_2_
	2.00, dd (12.0, 2.4)	
4		157.0, C
5	6.12, s	130.8, CH
6		196.3, C
7	6.28, s	125.6, CH
8		159.0, C
9	3.80, q (9.6)	33.4, CH
10	2.10, t (11.2)	30.4, CH_2_
	1.41, dd (11.2,8.4)	
11		40.7, C
12	3.29, s	70.6, CH_2_
13	1.02, s	18.7, CH_3_
14	2.03, s	25.8, CH_3_
15	4.52, d (16.5)	63.5, CH_2_
	4.22, dd (16.5, 2.0)	

### Computation Section

Systematic conformational analyses were performed via the Molecular Operating Environment (MOE) ver. 2009.10. (Chemical Computing Group, Canada) software package using the MMFF94 molecular mechanics force field calculation. The MMFF94 conformational analyses were further optimized using DFT at the B3LYP/6-311G(2d,p) basis set level. The stationary points have been checked as the true minima of the potential energy surface by verifying they do not exhibit vibrational imaginary frequencies. The 80 lowest electronic transitions were calculated at the B3LYP/6-311G(2d,p) level, and the rotational strengths of each electronic excitation were given using both dipole length and dipole velocity representations. ECD spectra were stimulated using a Gaussian function with a half-bandwidth of 0.3 eV. Equilibrium populations of conformers at 298.15 K were calculated from their relative free energies (ΔG) using Boltzmann statistics. The overall ECD spectra were then generated according to Boltzmann weighting of each conformer. The systematic errors in the prediction of the wavelength and excited-state energies are compensated for by employing UV correction.

### The 3-(4,5-Dimethylthiazol-2-yl)-5-(3-Carboxymethoxyphenyl)-2-(4-Sulfophenyl)-2H- Tetrazolium, Inner Salt (MTS) Assay

In a 96-well plate, each well was plated with (2–5) × 10^3^ cells (depending on the cell multiplication rate). After cell attachment overnight, the medium was removed, and each well was treated with 100 μL of medium containing 0.1% DMSO, or appropriate concentrations of the test compounds and the positive control cisplatin (100 mM as stock solution of a compound in DMSO and serial dilutions; the test compounds showed good solubility in DMSO and did not precipitate when added to the cells). The plate was incubated at 37°C for 48 h in a humidified, 5% CO_2_ atmosphere. Proliferation was assessed by adding 20 μL of MTS (Promega) to each well in the dark, followed by incubation at 37°C for 90 min. The assay plate was read at 490 nm using a microplate reader. The assay was run in triplicate.

### Antibacterial Assay

Antibacterial activities of compounds **1**–**8** were evaluated in replicate as per National Center for Clinical Laboratory Standards recommendations using broth micro dilution method to determine the MIC values with some modifications. In brief, the bacteria were grown in a LB medium (0.5% yeast extract, 1% peptone, 0.5% NaCl in deionized H_2_O). The assay was carried out in flat bottom 96-well microtiter plates. Microorganisms were pre-incubated at 37°C for 24 h in medium. Compounds **1**–**8** were dissolved in DMSO at an initial concentration at 25 mg/mL, and then 1 μL was added to 149 μL medium in 96-well microtiter plates. Then the microorganism solution was added into the 96-well plate (100 μL per well). The densities of the cells were approximately 1.0 × 10^6^ CFU/mL. Positive control drugs were ampicillin (Sigma, purity > 900 μg/mg) for *S. aureus* and *S. pneumoniae*, gentamicin (Sigma, purity ≥ 99%) for *E. coli* and *B. subtilis*. After 24 h incubation, the absorbance was determined at 600 nm by a microplate reader. The MIC value was determined as the lowest concentration inhibiting microbial growth.

## Results and Discussion

### Structure Elucidation

**FIGURE 2:**
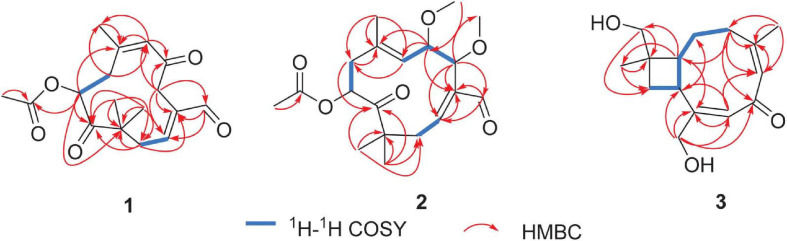
^1^H-^1^H COSY and HMBC correlations of compounds **1–3**.

**FIGURE 3:**
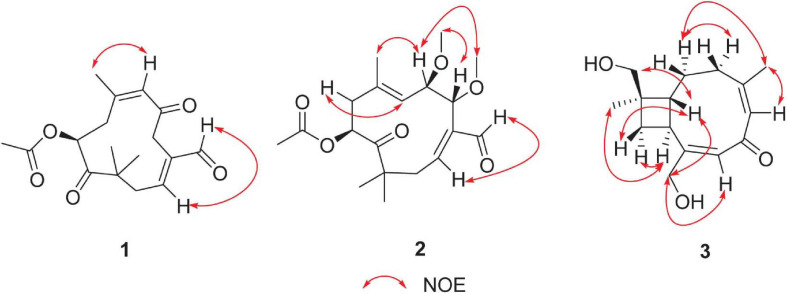
Selected NOESY correlations for compounds **1–3**.

**FIGURE 4:**
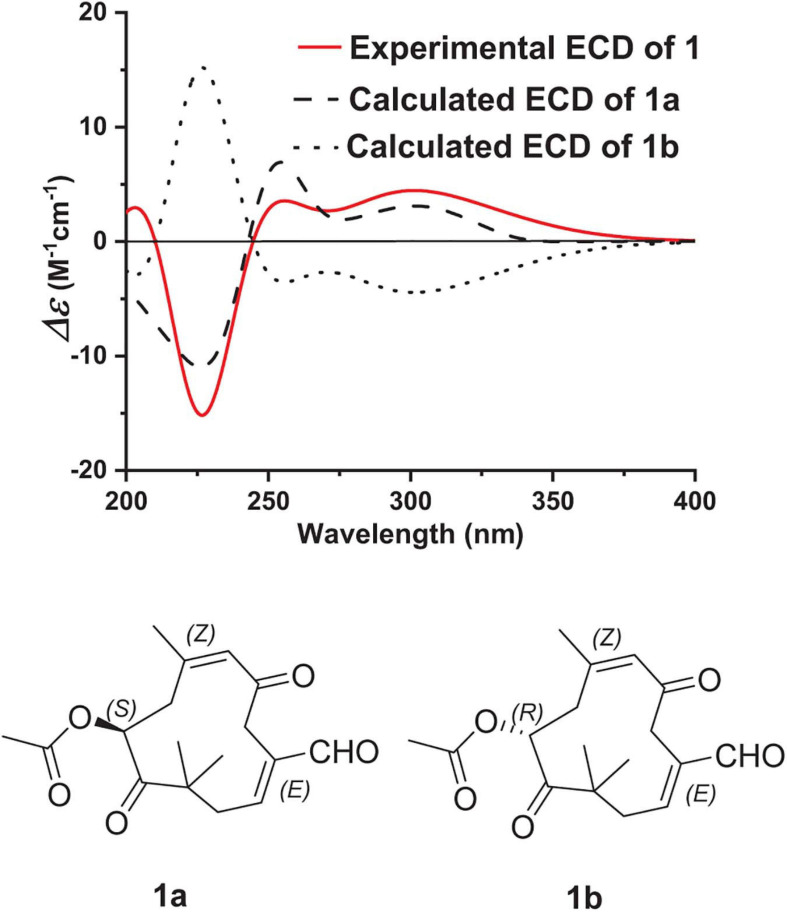
Experimental ECD spectrum of **1** in MeOH and the calculated ECD spectra of **1a** and **1b**.

Pestalothenin A (**1**) was obtained as a yellow powder. Its molecular formula was established as C_1__7_H_2__2_O_5_ on the basis of HRESIMS spectrum at *m/z* 329.1373 [M + Na]^+^ (calcd for C_1__7_H_2__2_O_5_Na, 329.1365), indicating 7° of unsaturation. Analysis of the ^1^H, ^13^C (Table 1 and Supplementary Figures 1, 2) and HSQC NMR (Supplementary Figure 4) spectroscopic data of **1** revealed the presence of four singlet methyl groups, three methylenes, one oxymethine, one sp^3^ quarternary carbon, two trisubstituted olefin units, one carboxylic carbon (δ_*C*_ 170.5), one aldehyde group (δ_*C*_ 193.2; δ_*H*_ 9.47) and two ketone carbons (δ_*C*_ 203.3 and 209.5, respectively), which accounted for 6° of unsaturation. Therefore, the remaining one unsaturation unit required that compound **1** possessed a monocyclic ring system. In the ^1^H–^1^H COSY spectrum (Figure 2 and Supplementary Figure 3), homonuclear vicinal coupling correlations between H_2_-1 and H-2 and between H_2_-8 and H-9 confirmed the structural fragments of C-1–C-2 and C-8–C-9 in **1**. In the HMBC spectrum (Figure 2 and Supplementary Figure 5), correlations from H-4 to C-2, C-3 and C-14 and from H-14 to C-2, C-3 and C-4 indicated that both C-4 and the aldehyde carbon C-14 (δ_*C*_ 193.2) were directly connected to the C-2/C-3 olefin at C-3. The HMBC crosspeaks (Figure 2 and Supplementary Figure 5) from H_2_-1 to C-11, C-12 and C-13, and from the geminal methyl groups H_3_-12 and H_3_-13 to C-1, the ketone carbon C-10 (δ_*C*_ 209.5) and the sp^3^ quarternary carbon C-11 (δ_*C*_ 47.3) indicated that C-1, C-10, C-12 and C-13 were all attached to C-11. Futhermore, HMBC correlations (Figure 2 and Supplementary Figure 5) from H_2_-8 to C-10 and from H-9 to C-10 and C-11 implied that the ketone carbon C-10 was located between C-9 and C-11. While the cross-peaks from H-9 and H_3_-17 to the carboxylic carbon C-16 (δ_*C*_ 170.5) established the location of the acetyl group at C-9. Other HMBC correlations (Figure 2 and Supplementary Figure 5) from the olefinic proton H-6 to C-5, C-7, C-8 and C-15, from H_2_-8 to C-6, C-7 and C-15, and from H_3_-15 to C-6, C-7 and C-8 led to the completion of C-5–C-6–C-7–C-8 subunit with the methyl group C-15 attached to the C-6/C-7 olefin at C-7. Key HMBC correlations (Figure 2 and Supplementary Figure 5) from H_2_-4 to C-5 and C-6 linked C-4 to the ketone carbon C-6, completing an 11-membered carbocyclic ring of compound **1**. Thus the planar structure of **1** was determined as a humulane-type sesquiterpenoid as shown. In the NOESY (Figure 3 and Supplementary Figure 6) spectrum, the cross peak between H-2 and H-14 assigned the C-2/C-3 olefin as *E-*geometry. While the C-6/C-7 olefin of **1** was assigned as *Z*-geometry on the basis of NOESY (Figure 3 and Supplementary Figure 6) correlation of H-6 with H_3_-15. To determine the absolute configuration of **1**, a comparison between the experimental and the simulated electronic circular dichroism (ECD) spectra (Figure 4) generated by the time-dependent density functional theory (TDDFT) (Zhu et al., 2019) for two enantiomers (9*S*)-**1** (**1a**) and (9*R*)-**1** (**1b**) was performed (Figure 4). The MMFF94 conformational search and DFT re-optimization at the B3LYP/6-311G(2d,p) level yielded two lowest energy conformers for **1a** (Supplementary Figure 19). The experimental ECD curve of **1** was nearly identical to the calculated ECD spectrum of **1a**, suggested the 9*S* absolute configuration for **1**.

**FIGURE 5:**
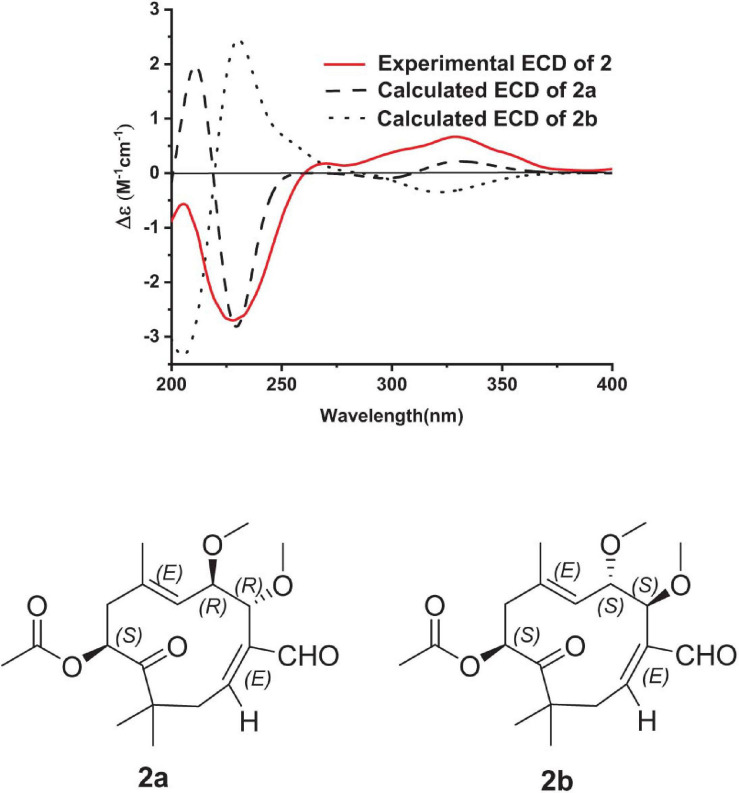
Experimental ECD spectrum of **2** in MeOH and the calculated ECD spectra of **2a** and **2b**.

The molecular formula of pestalothenin B (**2**) was deduced as C_1__9_H_2__8_O_6_ (6° of unsaturation) by analysis of HRESIMS spectrum at *m/z* 375.1882 [M + Na]^+^ (calcd for C_1__9_H_2__8_O_6_Na, 375.1886) and NMR data. Analysis of its NMR spectroscopic data (Table 1 and Supplementary Figures 7, 8, 10) revealed the presence of six methyl groups (two methoxy groups), two methylenes, three oxymethines, one sp^3^ quaternary carbon, two trisubstituted olefin units, one carboxylic carbon (δ_*C*_ 170.0), one aldehyde group (δ_*C*_ 194.0; δ_*H*_ 9.37) and one ketone carbon (δ_*C*_ 208.3). These data were similar to those of **1**, indicating that compound **2** was also a humulane-type sesquiterpene. The main differences were that the methylene group at C-4 (δ_*C*__/H_ 39.4/2.85, 3.83) and the ketone carbon C-5 (δ_*C*_ 203.3) in **1** were replaced by two oxymethines (δ_*C*__/H_ 79.2/4.71; 78.9/4.14) and two methoxy groups (δ_*C*__/H_ 57.2/3.26; 56.7/3.24) in **2**. These assignments were further confirmed by 2D NMR data analysis. In the ^1^H-^1^H COSY spectrum (Figure 2 and Supplementary Figure 9), the cross-peaks of H-4 with H-5 and H-5 with H-6 indicated a C-4–C-5–C-6 subunit of **2**. HMBC correlations (Figure 2 and Supplementary Figure 11) from H-4 to C-2, C-3, C-14 and C-15, from H-5 to C-3, C-7 and C-16, from H_3_-15 to C-4 and from H_3_-16 to C-5 linked C-4 to C-3 and located two methoxy groups at C-4 and C-5, respectively. Therefore, the planar structure of compound **2** was established as shown. The relative configuration of **2** was assigned by analysis of NOESY data. NOESY correlations (Figure 3 and Supplementary Figure 12) of H-2 with H-14 assigned the C-2/C-3 olefin as *E-*geometry. The C-6/C-7 olefin of **2** was also assigned as *E*-geometry based on NOESY correlations of H-6 with H-8 and H-5 with H_3_-17. NOESY correlation (Figure 3 and Supplementary Figure 12) of H-5 with H_3_-15 indicated the assignment of the α-configuration of H-5 and H_3_-15. While NOESY correlation (Figure 3 and Supplementary Figure 12) of H-4 with H_3_-16 indicated that H-4 and H_3_-16 were in the β-orientation, thus establishing the relative configuration of **2**. The absolute configuration of C-9 in **2** was deduced as 9*S* on the basis of biosynthetic considerations and by analogy to **1**, **4**, and **5** (Liao et al., 2013; Kapustina et al., 2020). The absolute configurations of C-4 and C-5 in **2** were also deduced by comparison of the experimental and calculated ECD spectra for the two stereoisomers, (4*R*, 5*R*, 9*S*)-**2** (**2a**) and (4*S*, 5*S*, 9*S*)-**2** (**2b**) (Figure 5). The MMFF94 conformational search and DFT re-optimization at the B3LYP/6-311G(2d,p) level yielded 3 lowest energy conformers for **2a** (Supplementary Figure 19). The overall calculated ECD spectra of **2a** and **2b** were then generated by Boltzmann weighting of the conformers (Figure 5). The experimental CD curve of **2** matched well with the calculated ECD spectrum of **2a**, suggesting that compound **2** has the absolute configuration of 4*R*, 5*R*, 9*S.*

**FIGURE 6:**
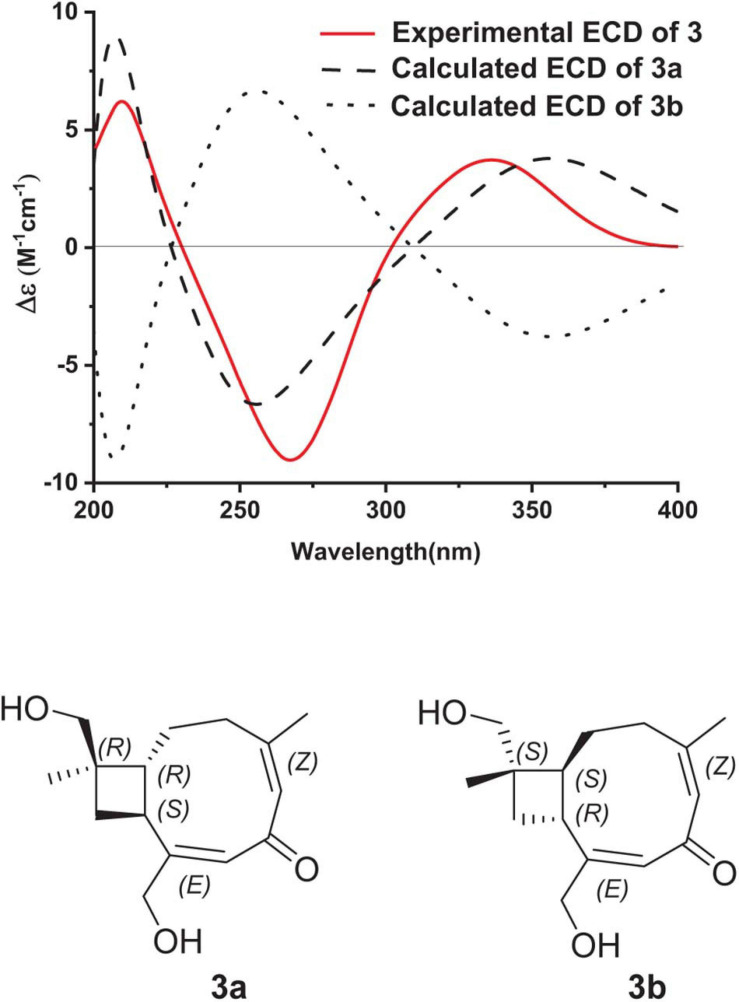
Experimental ECD spectrum of **3** in MeOH and the calculated ECD spectra of **3a** and **3b**.

Pestalothenin (**3**) was determined to have the molecular formula C_1__5_H_2__2_O_3_ based on HRESIMS data spectrum at *m/z* 251.1665 [M + H]^+^ (calcd for C_1__5_H_2__3_O_3_, 251.1642) with 5° of unsaturation. Analysis of NMR data (Table 2 and Supplementary Figures 13, 14, 16) of **1** revealed the presence of two methyls, five methylenes (two oxygenated), two methines, one sp^3^ quaternary carbon, two trisubstituted olefin units, and one α,β-unsaturated ketone carbon (δ_*C*_ 196.3). These data accounted for all ^1^H and ^13^C resonances except for two exchangeable protons, and suggested that **3** was a bicyclic compound. Analysis of the ^1^H–^1^H COSY spectrum (Figure 2 and Supplementary Figure 15) of **3** showed one isolated spin-system of C-3–C-2–C-1–C-9–C-10, as shown by the bold bonds in Figure 2. HMBC correlations (Figure 2 and Supplementary Figure 17) from H_2_-12 and H_3_-13 to C-1, C-10, and C-11, from H-1 and H_2_-10 to C-11, C-12 and C-13 allowed the construction of the cyclobutane ring. Further HMBC crosspeaks (Figure 2 and Supplementary Figure 17) from H_2_-3 to C-4, C-5, and C-14, from the olefinic proton H-5 to C-3, C-4 and C-14, and from H_3_-14 to C-3, C-4 and C-5 indicated that both C-3 and C-14 were directly connected to the C-4/C-5 olefin at C-4. Other correlations (Figure 2 and Supplementary Figure 17) from the olefinic proton H-7 to C-8, C-9 and C-15, from H-9 to C-7, C-8 and C-15, and from H_2_-15 to C-7, C-8 and C-9 completed the C-7–C-8–C-9 subunit with C-15 attached to the C-7/C-8 olefin at C-8. Finally, HMBC correlations (Figure 2 and Supplementary Figure 17) from H-5 to C-7, from H-7 to C-5, and from H_2_-15 and H-3 to the α,β-unsaturated ketone carbon C-6 (δ_*C*_ 196.3) revealed that C-6 was located between C-5 and C-7 to form the cyclononene ring, which was fused to the cyclobutane ring at C-1/C-9 to complete the bicyclo[7.2.0]undeca-2,5-dien-4-one core structure of **3**. The two exchangeable protons were located at C-12 and C-15, respectively, by default, which partially supported by the chemical shift values for C-12 (δ_*C*_ 70.6) and C-15 (δ_*C*_ 63.5). Thus, the gross structure of **3** was established as a caryophyllene-type sesquiterpenoid (Figure 1). The relative configuration of **3** was established on the basis of the NOESY data (Figure 4). NOESY correlations (Figure 3 and Supplementary Figure 18) of H-5 with H_3_-14, and of H-7 with H_2_-15 defined the *Z*-geometry and *E*-geometry for C-4/C-5 and C-7/C-8 olefins, respectively. NOESY correlations (Figure 3 and Supplementary Figure 18) of H-1 with H-10, H_2_-12 and H_2_-15 indicated that these protons were on the same side of the ring system, whereas those of H-9 with H-10 and H_3_-13 placed these protons on the opposite side of the molecule, thus establishing the relative configuration of **3**. To establish the absolute configuration of **3**, ECD spectrum of **3** was recorded in MeOH and compared with the DFT-calculated spectra of two enantiomers 1*R*, 9*S*, 11*R* and 1*S*, 9*R*, 11*S* at the B3LYP/6-311 + G(2d,p) level. The MMFF94 conformational search and DFT re-optimization at the B3LYP/6-311G(2d,p) level yielded 3 lowest energy conformers for **3a** (Supplementary Figure 19). The calculated ECD spectrum of **3a** showed a good agreement with the experimental curve (Figure 6), which supported the absolute configuration being 1*R*, 9*S*, 11*R*. Thus, the completed structure of **3** was elucidated as depicted in Figure 1.

On the basis of the NMR and MS spectroscopic data comparison with those reported in the literatures, in addition to the specific rotation, the other five compounds were identified as 14-acetylhumulane (**4**) (Liu et al., 2016b), 9,15-Dihydroxy-2,6-humuladiene-5,10-dione (**5**) (Pulici et al., 1996a), punctaporonin H (**6**) (Wu et al., 2014), pestalotiopsin E (**7**) (Xiao et al., 2017), and pestalotiopsin C (**8**) (Pulici et al., 1997; Figure 1).

### Biological Activity

Compounds **1**–**8** were evaluated for antibacterial activity against *Staphylococcus aureus* (CGMCC 1.2465), *Bacillus subtilis* (ATCC 6633), *Streptococcus pneumoniae* (CGMCC 1.1692), and *Escherichia coli* (CGMCC 1.2340). However, none of these compounds showed antibacterial activities toward these bacteria (MIC > 50 μg/mL). Compounds **1**–**8** were tested for cytotoxicity against a panel of five human tumor cell lines, A549 (human lung adenocarcinoma cell line), T24 (human bladder carcinoma cell line), HeLa (human cervical carcinoma cell line), MCF-7 (human breast cancer cell line) and HepG2 (human hepatoma cell line). Compound **6** showed cytotoxic to T24 and MCF-7 cell lines, with IC_50_ values of 45.7 and 37.6 μM, respectively, whereas the corresponding positive control cisplatin showed IC_50_ values of 8.4 and 10.5 μM, respectively. While compounds **1**–**5**, **7**, and **8** did not show detectable inhibitory effects on the cell lines tested at 50 μM.

## Conclusion

In summary, eight sesquiterpeniods including three new ones, pestalothenins A–C (**1**–**3**) were identified from the fermentation of the plant endophytic fungus *P. theae* (N635). The structures of the new compounds were elucidated via analyses of their MS, NMR, and ECD spectroscopic data. Pestalothenin A (**1**) differs from the known fungal metabolite 9,15-Dihydroxy-2,6-humuladiene-5,10-dione (**5**) (Pulici et al., 1996a) by different configuration of C-2/C-3 and C-6/C-7 olefins and by having aldehyde and acetyl groups instead of hydroxymethyl and the hydroxy groups at C-3 and C-9, respectively. Pestalothenin B (**2**) is structurally related to pestalothenin A, but differs in having *E*-geometry for C-6/C-7 olefin and having two oxymethines and two methoxy groups rather than the methylene and ketone groups at C-4 and C-5, respectively. Biogenetically, the humulane-type sesquiterpeniods (**1**, **2**, **4,** and **5**) could be derived from humulene which was formed from farnesyl pyrophosphate, first via oxidation, reduction and dehydration, and then followed by a series of methylation and acetylation reactions. While pestalothenin C (**3**) differs from the known humifusane A (Tian et al., 2011) by having oxymethylene group instead of methyl group at C-8. Biogenetically, humulene could be the biosynthetic intermediator of β-caryophyllene which acted as a key precursor in nature to form diverse tricyclic sesquiterpenes by transannular cyclizations. Starting from β-caryophyllene, caryophyllene-type sesquiterpeniods (**3** and **6**–**8**) could be generated via a series of reactions including transannular cyclization, oxidation, reduction, methylation and acetylation reactions. Compound **6** showed cytotoxic against T24 and MCF-7 cell lines. Our findings not only expand the chemical space of humulane-type and caryophyllene-type sesquiterpeniods, but also suggest that the fungal genus *Pestalotiopsis* might be a rich source of bioactive secondary metabolites.

## Data Availability Statement

The datasets presented in this study can be found in online repositories. The names of the repository/repositories and accession number(s) can be found in the article/Supplementary Material.

## Author Contributions

LL designed the experiments. GL and RH performed the experiments. GL, YZ, and LL wrote and revised the manuscript. All authors contributed to the article and approved the submitted version.

## Conflict of Interest

The authors declare that the research was conducted in the absence of any commercial or financial relationships that could be construed as a potential conflict of interest.

## References

[B1] Ayer W. A. Browne L. M. (1981). Terpenoid metabolites of mushrooms and related basidiomycetes. *Tetrahedron Lett.* 37 2199–2248. 10.1016/S0040-4020(01)97979-7

[B2] Bardón A. Kamiya N. Toyota M. Takaoka S. Asakawa Y. (1999). Sesquiterpenoids, hopanoids and bis (bibenzyls) from the Argentine liverwort *Plagiochasma rupestre*. *Phytochemistry* 52 1323–1329. 10.1016/S0031-9422(99)00452-5

[B3] Chen Z. M. Fan Q. Y. Yin X. Yang X. Y. Li Z. H. Feng T. (2014). Three new humulane sesquiterpenes from cultures of the fungus *Antrodiella albocinnamomea*. *Nat. Prod. Bioprospect.* 4 207–211. 10.1007/s13659-014-0032-4 25089238 PMC4111878

[B4] Collado I. G. Aleu J. Macías S. Antonio J. Hernández G. R. (1994). Inhibition of botrytzs cznerea by new sesquiterpenoid compounds obtained from the rearrangement of isocaryophyllene. *J. Nat. Prod.* 57 738–746. 10.1021/np50108a009

[B5] Daniewsk W. M. Grieco P. A. Huffman J. C. RymkiewLcz A. Wawrzun A. (1981). Isolation of 12-hydroxycaryophyllene-4,5-oxide, a sesquiterpene from *lactarius camphoratus*. *Phytochemistry* 20 2733–2734. 10.1016/0031-9422(81)85276-4

[B6] Fidyt K. Fiedorowicz A. Strza̧dała L. Szumny A. (2016). β-caryophyllene and β-caryophyllene oxide-natural compounds of anticancer and analgesic properties. *Cancer Med. US* 5 3007–3017. 10.1002/cam4.816 27696789 PMC5083753

[B7] Fraga B. M. (2011). Natural sesquiterpenoids. *Nat. Prod. Rep.* 28 1580–1610. 10.1039/c1np00046b 21808787

[B8] Fraga B. M. (2013). Natural sesquiterpenoids. *Nat. Prod. Rep.* 30 1226–1264. 10.1039/c3np70047j 23884176

[B9] Ghalib R. M. Hashim R. Sulaiman O. Mehdi S. H. Valkonen A. Rissanen K. (2012). A novel caryophyllene type sesquiterpene lactone from *Asparagus falcatus* (Linn.); Structure elucidation and anti-angiogenic activity on HUVECs. *Eur. J. Med. Chem*. 47 601–607. 10.1016/j.ejmech.2011.10.037 22074984

[B10] Guo L. F. Lin J. Niu S. Liu S. B. Liu S. C. Liu L. (2020a). Pestalotiones A-D: four new secondary metabolites from the plant endophytic fungus *Pestalotiopsis theae*. *Molecules* 25:470. 10.3390/molecules25030470 31979166 PMC7037426

[B11] Guo L. F. Liu G. R. Liu L. (2020b). Caryophyllene-type sesquiterpenoids and α-furanones from the plant endophytic fungus *Pestalotiopsis theae*. *Chin. J. Nat. Med.* 18 261–267. 10.1016/s1875-5364(20)30032-7 32402402

[B12] Han H. Guo Z. K. Zhang B. Zhang M. Shi J. Li W. (2019). Bioactive phenazines from an earwig-associated *Streptomyces* sp. *Chin. J. Nat. Med.* 17 475–480. 31262460 10.1016/S1875-5364(19)30055-X

[B13] Hwang I. H. Swenson D. C. Gloer J. B. Wicklow D. T. (2015). Pestaloporonins: caryophyllene-derived sesquiterpenoids from a fungicolous isolate of *Pestalotiopsis* sp. *Org. Lett.* 17 4284–4287. 10.1021/acs.orglett.5b02080 26287562

[B14] Jiang C. S. Zhou Z. F. Yang X. H. Lan L. F. Gu Y. C. Ye B. P. (2018). Antibacterial sorbicillin and diketopiperazines from the endogenous fungus *Penicillium* sp. GD6 associated Chinese mangrove *Bruguiera gymnorrhiza*. *Chin. J. Nat. Med.* 16 358–365. 10.1016/s1875-5364(18)30068-2 29860997

[B15] Kapustina I. I. Makarieva T. N. Guzii A. G. Kalinovsky A. I. Popov R. S. Dyshlovoy S. A. (2020). Leptogorgins A-C, Humulane sesquiterpenoids from the vietnamese gorgonian *Leptogorgia* sp. *Mar. Drugs* 18 310–320. 10.3390/md18060310 32545757 PMC7344390

[B16] Liao C. S. Tang C. P. Yao S. Ye Y. (2013). Humulane-type sesquiterpenoids from *Pilea cavaleriei* subsp. *crenata*. *Org. Biomol. Chem.* 11 4840–4846. 10.1039/c3ob40872h 23764729

[B17] Liu L. Han Y. Xiao J. H. Li L. Guo L. D. Jiang X. J. (2016a). Chlorotheolides A and B, spiroketals generated via Diels-Alder reactions in the endophytic fungus *Pestalotiopsis theae*. *J. Nat. Prod.* 79 2616–2623. 10.1021/acs.jnatprod.6b00550 27731995

[B18] Liu L. Li Y. Liu S. C. Zheng Z. H. Chen X. L. Zhang H. (2009). Chloropestolide A, an antitumor metabolite with an unprecedented spiroketal skeleton from *Pestalotiopsis fici*. *Org. Lett.* 11 2836–2839. 10.1021/ol901039m 19496604

[B19] Liu L. Niu S. B. Lu X. H. Chen X. L. Zhang H. Guo L. D. (2010). Unique metabolites of *Pestalotiopsis fici* suggest a biosynthetic hypothesis involving a Diels–Alder reaction and then mechanistic diversification. *Chem. Comm.* 46 460–462. 10.1039/b918330b 20066325

[B20] Liu M. T. He Y. Shen L. Hu Z. X. Zhang Y. H. (2019). Bipolarins A–H, eight new ophiobolin-type sesterterpenes with antimicrobial activity from fungus *Bipolaris* sp. TJ403-B1. *Chin. J. Nat. Med.* 17 935–944. 10.1016/s1875-5364(19)30116-5 31882049

[B21] Liu Y. Yang M. H. Wang X. B. Li T. X. Kong L. Y. (2016b). Caryophyllene sesquiterpenoids from the endophytic fungus, *Pestalotiopsis* sp. *Fitoterapia* 109 119–124. 10.1016/j.fitote.2015.12.003 26687557

[B22] Luo D. Q. Gao Y. Gao J. M. Wang F. Yang X. L. Liu J. K. (2006). Humulane-type sesquiterpenoids from the mushroom *Lactarius mitissimus*. *J. Nat. Prod.* 69 1354–1357. 10.1016/j.bcp.2008.05.019 16989534

[B23] Luo X. W. Lin Y. Lu Y. J. Zhou X. F. Liu Y. H. (2019). Peptides and polyketides isolated from the marine sponge-derived fungus *Aspergillus terreus* SCSIO 41008. *Chin. J. Nat. Med.* 17 149–154. 30797421 10.1016/S1875-5364(19)30017-2

[B24] Petrini O. Sieber T. N. Toti L. Viret O. (1992). Ecology, metabolite production, and substrate utilization in endophytic fungi. *Nat. Toxins* 1 185–196. 10.1002/nt.2620010306 1344919

[B25] Pulici M. Sugawar F. Koshino H. Okada G. Esumi Y. Uzawa J. (1997). Metabolites of *pestalotiopsis* spp., endophytic fungi of *taxus brevifolia*. *Phytochemistry* 46 313–319. 10.1016/S0031-9422(97)00285-9

[B26] Pulici M. Sugawara F. Koshino H. Uzawa J. Yoshida S. (1996a). Metabolites of endophytic fungi of *Taxus brevifolia*: the first highly functionalized humulane of fungal origin. *J. Chem. Res. Synop.* 8 378–379. 10.1016/0925-8388(96)02333-X

[B27] Pulici M. Sugawara F. Koshino H. Uzawa J. Yoshida S. (1996b). Pestalotiopsins A and B: new caryophyllenes from an endophytic fungus of *Taxus brevifolia*. *J. Org. Chem.* 61 2122–2124.

[B28] Strobel G. Daisy B. Castillo U. Harper J. (2004). Natural products from endophytic microorganisms. *J. Nat. Prod.* 67 257–268. 10.1021/np030397v 14987067

[B29] Strobel G. A. (2003). Endophytes as sources of bioactive products. *Microbes Infect.* 5 535–544. 10.1016/s1286-4579(03)00073-X 12758283

[B30] Takao K. i. Hayakawa N. Yamada R. Yamaguchi T. Morita U. Kawasaki S. (2008). Total synthesis of (-)-pestalotiopsin A. *Angew. Chem.* 120 3474–3477. 10.1002/anie.200800253 18366053

[B31] Takao K. i. Hayakawa N. Yamada R. Yamaguchi T. Saegusa H. Uchida M. (2009). Total syntheses of (+)- and (-)-pestalotiopsin A. *J. Org. Chem.* 74 6452–6461. 10.1021/jo9012546 19658390

[B32] Tian Y. Sun L. M. Li B. Liu X. Q. Dong J. X. (2011). New anti-HBV caryophyllane-type sesquiterpenoids from *Euphorbia humifusa* Willd. *Fitoterapia* 82 251–254. 10.1016/j.fitote.2010.10.005 20940034

[B33] Toyota M. Omatsu I. Braggins J. Asakawa Y. (2004). New humulane-type sesquiterpenes from the liverworts *Tylimanthus tenellus* and *Marchantia emarginata* subsp. *tosana*. *Chem. Pharm. Bull.* 52 481–484. 10.1002/chin.200437181 15056972

[B34] Wang J. He W. Kong F. Tian X. Wang P. Zhou X. (2017). Ochracenes A-I, humulane-derived sesquiterpenoids from the antarctic fungus *Aspergillus ochraceopetaliformis*. *J. Nat. Prod.* 80 1725–1733. 10.1021/acs.jnatprod.6b00810 28598633

[B35] Wu Z. Liu D. Proksch P. Guo P. Lin W. (2014). Punctaporonins H-M: caryophyllene-type sesquiterpenoids from the sponge-associated fungus *Hansfordia sinuosae*. *Mar. Drugs* 12 3904–3916. 10.3390/md12073904 24983636 PMC4113805

[B36] Wu Z. H. Liu D. Xu Y. Chen J. L. Lin W. H. (2018). Antioxidant xanthones and anthraquinones isolated from a marine-derived fungus *Aspergillus versicolor*. *Chin. J. Nat. Med.* 16 219–224. 10.1016/s1875-5364(18)30050-5 29576058

[B37] Xiao J. Lin L. B. Hu J. Y. Jiao F. R. Duan D. Z. Zhang Q. (2017). Highly oxygenated caryophyllene-type and drimane-type sesquiterpenes from *Pestalotiopsis adusta*, an endophytic fungus of *Sinopodophyllum hexandrum*. *RSC Adv.* 7 29071–29079. 10.1039/C7RA04267A

[B38] Zhu S. M. Ren F. X. Guo Z. Liu J. H. Liu X. Z. Liu G. (2019). Rogersonins A and B, imidazolone *N*-Oxide-incorporating indole alkaloids from a *verG* disruption mutant of *Clonostachys rogersoniana*. *J. Nat. Prod.* 82 462–468. 10.1021/acs.jnatprod.8b00851 30576135

