# Supplementary material for: New Bioactive Sesquiterpeniods From the Plant Endophytic Fungus Pestalotiopsis theae
Source: Front Microbiol. 2021 Mar 31;12:641504. doi: 10.3389/fmicb.2021.641504 (PMC8044550; doi:10.3389/fmicb.2021.641504)
Supplement: Supplementary file 1 [file Data_Sheet_1.docx]

Supplementary Material

| **Supplementary Figure 1.** ^1^H NMR spectrum of pestalothenin A (**1**; 500 MHz, CDCl_3_) | S3 |
| --- | --- |
| **Supplementary Figure 2.** ^13^C NMR spectrum of pestalothenin A (**1**; 125 MHz, CDCl_3_) | S4 |
| **Supplementary Figure 3.** ^1^H-^1^H COSY spectrum of pestalothenin A (**1**; 500 MHz, CDCl_3_) | S5 |
| **Supplementary Figure 4.** HSQC spectrum of pestalothenin A (**1**; 500 MHz, CDCl_3_) | S6 |
| **Supplementary Figure 5.** HMBC spectrum of pestalothenin A (**1**; 500 MHz, CDCl_3_) | S7 |
| **Supplementary Figure 6.** NOESY spectrum of pestalothenin A (**1**; 500 MHz, CDCl_3_) | S8 |
| **Supplementary Figure 7.** ^1^H NMR spectrum of pestalothenin B (**2**; 600 MHz, CDCl_3_) | S9 |
| **Supplementary Figure 8.** ^13^C NMR spectrum of pestalothenin B (**2**; 150 MHz, CDCl_3_) | S10 |
| **Supplementary Figure 9.** ^1^H-^1^H COSY spectrum of pestalothenin B (**2**; 600 MHz, CDCl_3_) | S11 |
| **Supplementary Figure 10.** HSQC spectrum of pestalothenin B (**2**; 600 MHz, CDCl_3_) | S12 |
| **Supplementary Figure 11.** HMBC spectrum of pestalothenin B (**2**; 600 MHz, CDCl_3_) | S13 |
| **Supplementary Figure 12.** NOESY spectrum of pestalothenin B (**2**; 600 MHz, CDCl_3_) | S14 |
| **Supplementary Figure 13.** ^1^H NMR spectrum of pestalothenin C (**3**; 400 MHz, methanol-*d*_4_) | S15 |
| **Supplementary Figure 14.** ^13^C NMR spectrum of pestalothenin C (**3**; 100 MHz, methanol-*d*_4_) | S16 |
| **Supplementary Figure 15.** ^1^H-^1^H COSY spectrum of pestalothenin C (**3**; 400 MHz, methanol-*d*_4_) | S17 |
| **Supplementary Figure 16.** HSQC spectrum of pestalothenin C (**3**; 400 MHz, methanol-*d*_4_) | S18 |
| **Supplementary Figure 17.** HMBC spectrum of pestalothenin C (**3**; 400 MHz, methanol-*d*_4_) | S19 |
| **Supplementary Figure 18.** NOESY spectrum of pestalothenin C (**3**; 400 MHz, methanol-*d*_4_) | S20 |
| **Supplementary Figure 19.** ECD conformers of pestalothenins A–C (**1**–**3**) | S21 |
| **Supplementary Figure 20.** HRESIMS spectrum of pestalothenin A (**1**) | S24 |
| **Supplementary Figure 21.** HRESIMS spectrum of pestalothenin B (**2**) | S25 |
| **Supplementary Figure 22.** HRESIMS spectrum of pestalothenin C (**3**) | S26 |
| **Supplementary Figure 23.** Experimental ECD spectrum of pestalothenin A (**1**) in MeOH | S27 |
| **Supplementary Figure 24.** Experimental ECD spectrum of pestalothenin B (**2**) in MeOH | S28 |
| **Supplementary Figure 25.** Experimental ECD spectrum of pestalothenin C (**3**) in MeOH | S29 |
|  |  |


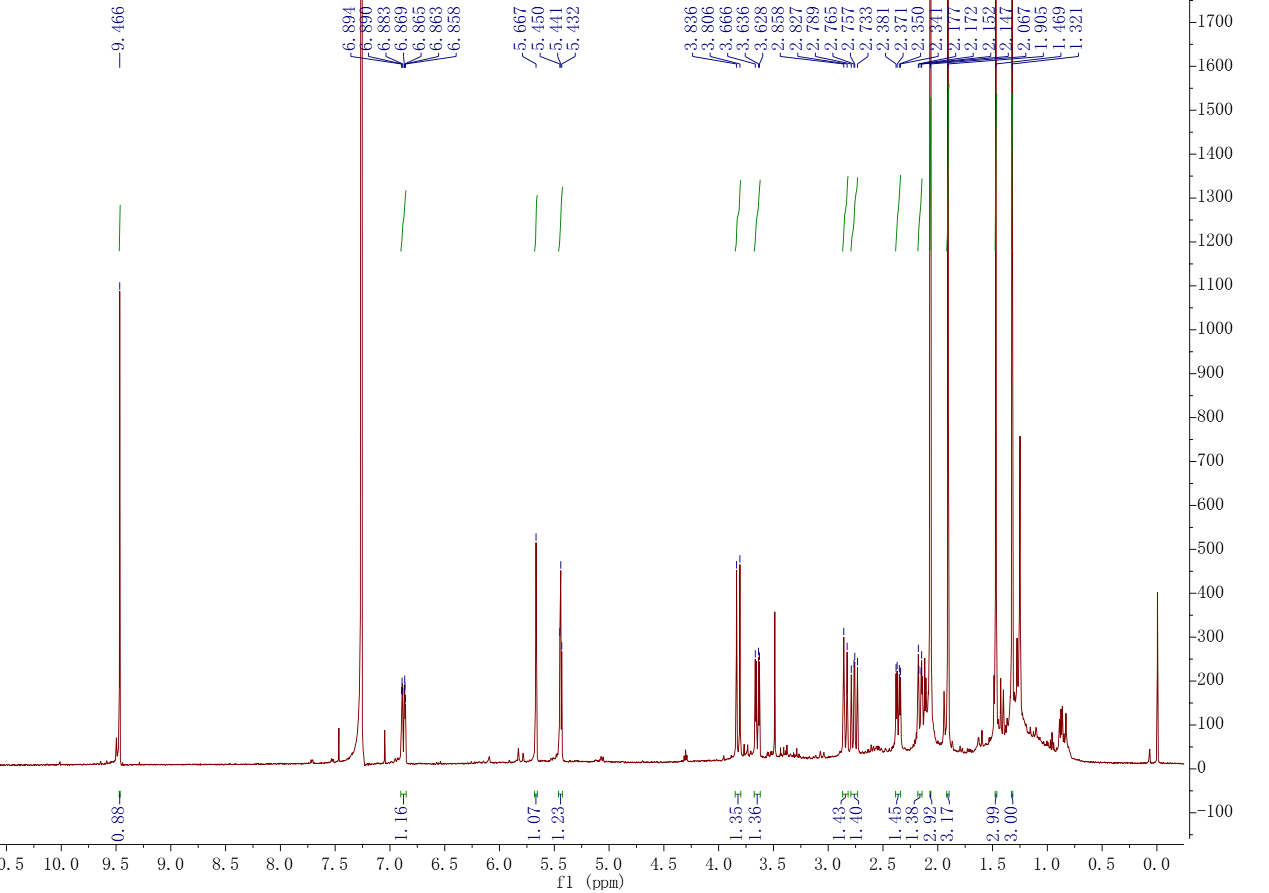


**Supplementary Figure 1.** ^1^H NMR spectrum of pestalothenin A (**1**; 500 MHz, CDCl_3_)


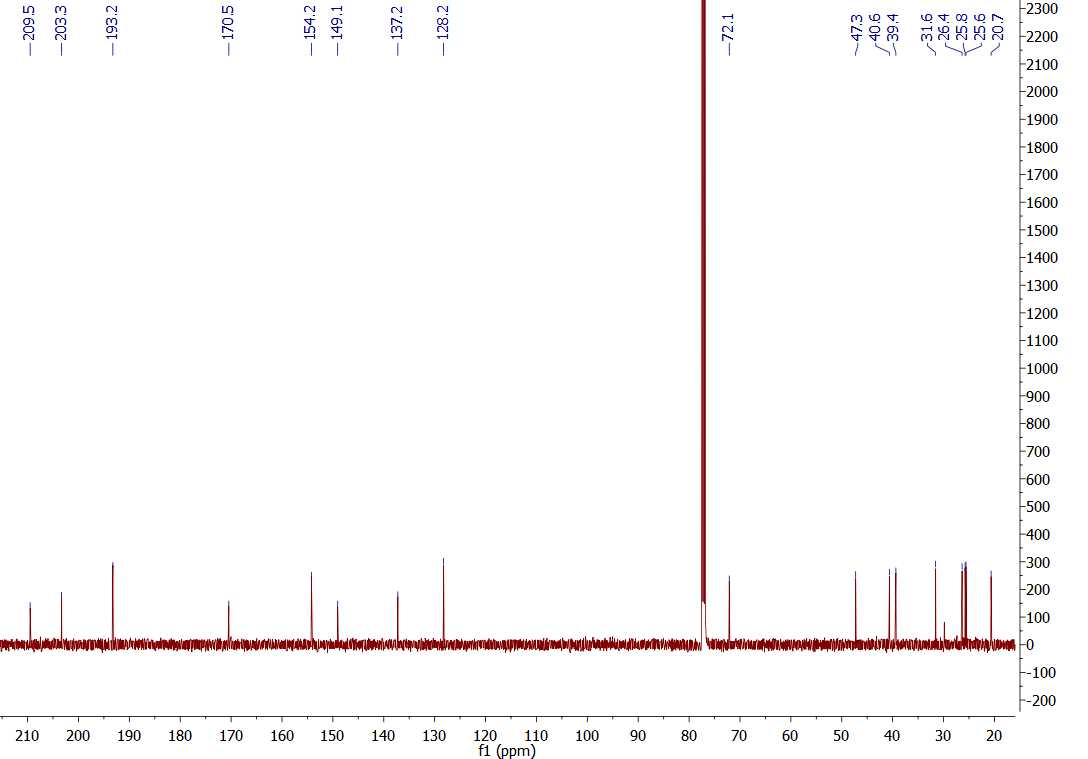


**Supplementary Figure 2.** ^13^C NMR spectrum of pestalothenin A (**1**; 125 MHz, CDCl_3_)


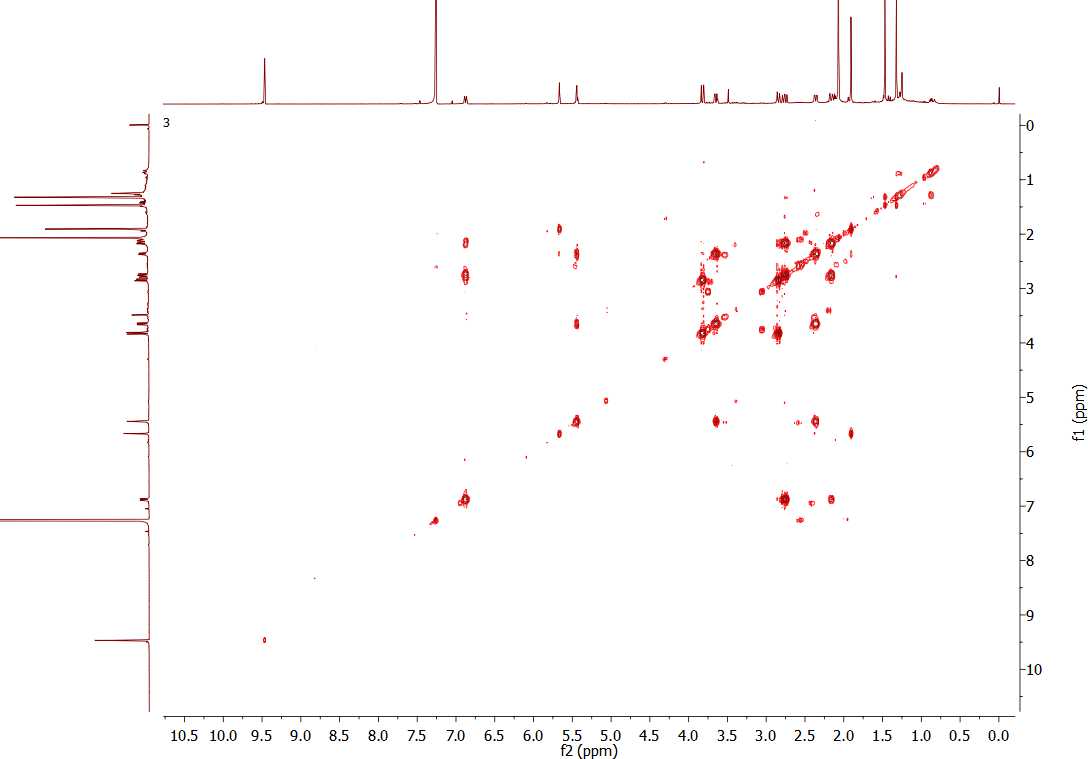


**Supplementary Figure 3.** ^1^H-^1^H COSY spectrum of pestalothenin A (**1**; 500 MHz, CDCl_3_)


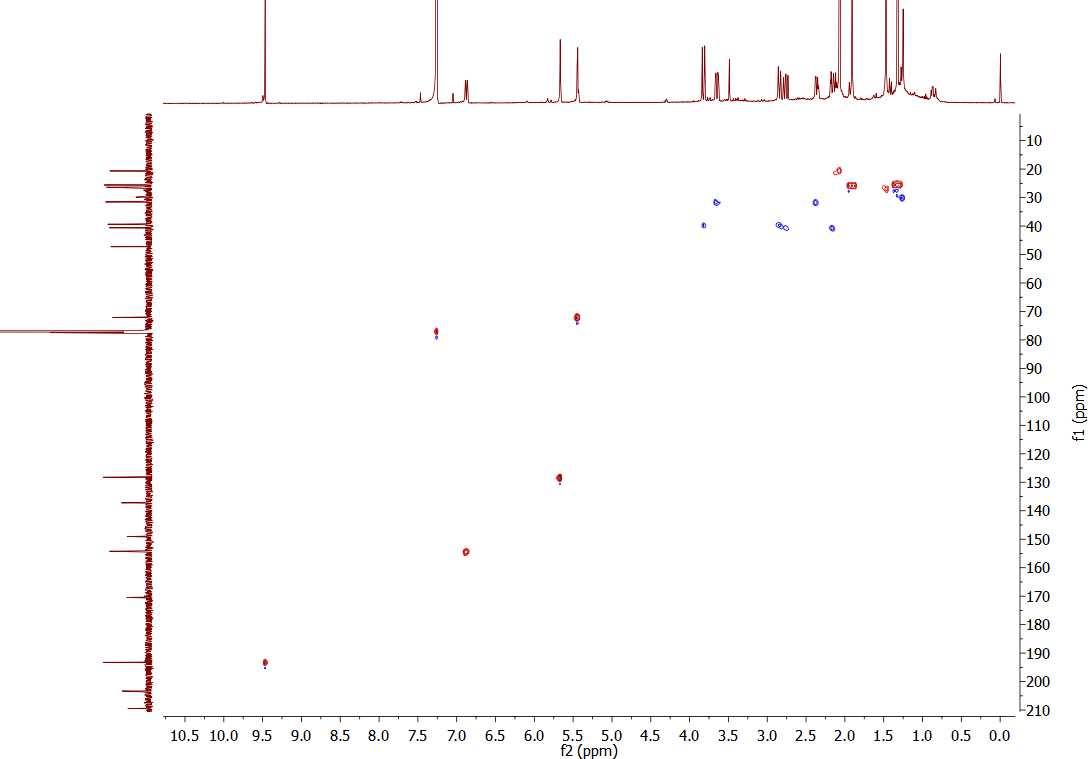


**Supplementary Figure 4.** HSQC spectrum of pestalothenin A (**1**; 500 MHz, CDCl_3_)


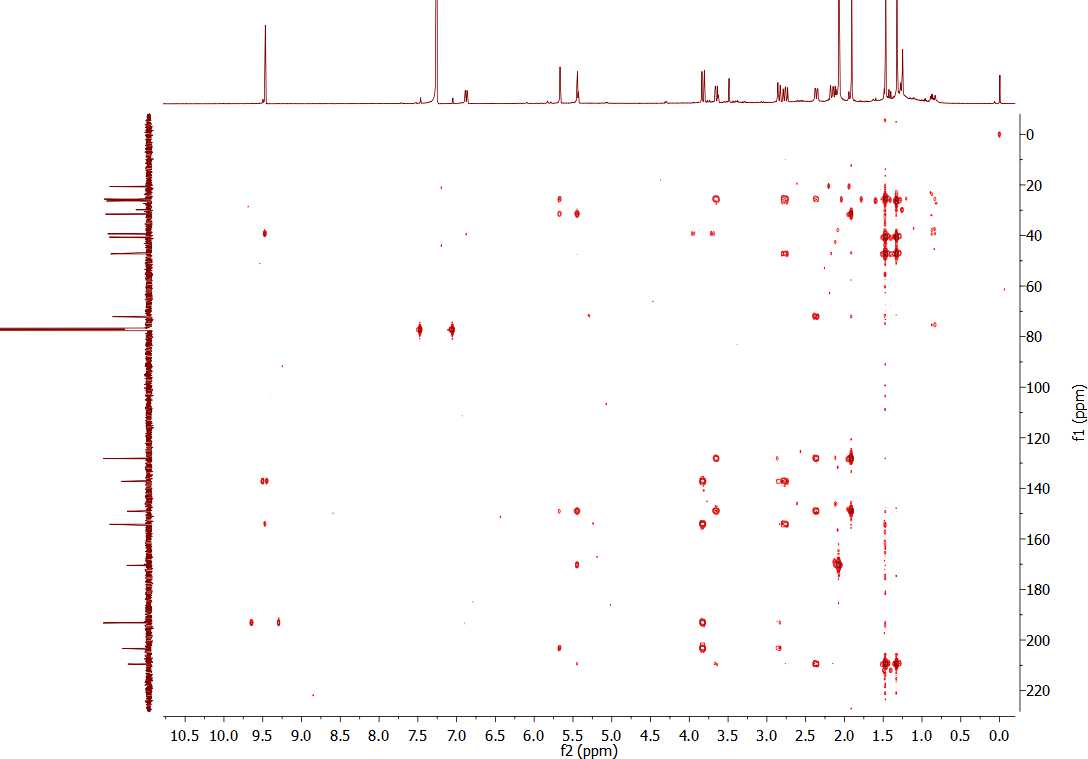


**Supplementary Figure 5.** HMBC spectrum of pestalothenin A (**1**; 500 MHz, CDCl_3_)


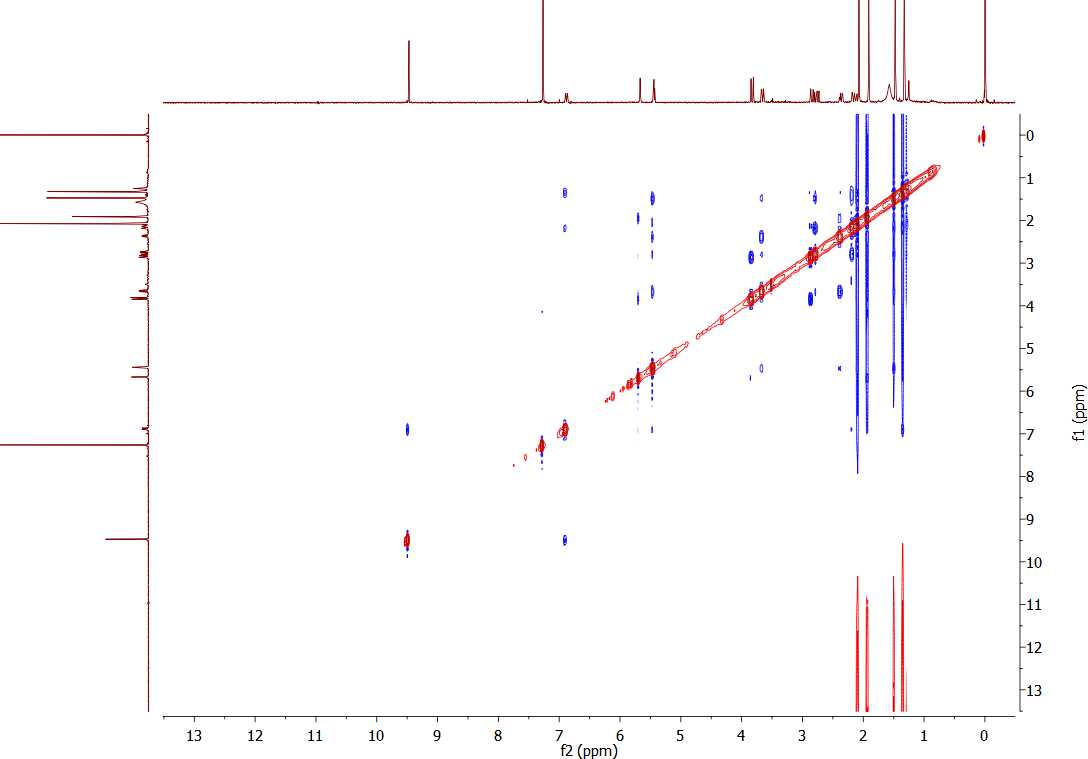


**Supplementary Figure 6.** NOESY spectrum of pestalothenin A (**1**; 500 MHz, CDCl_3_)


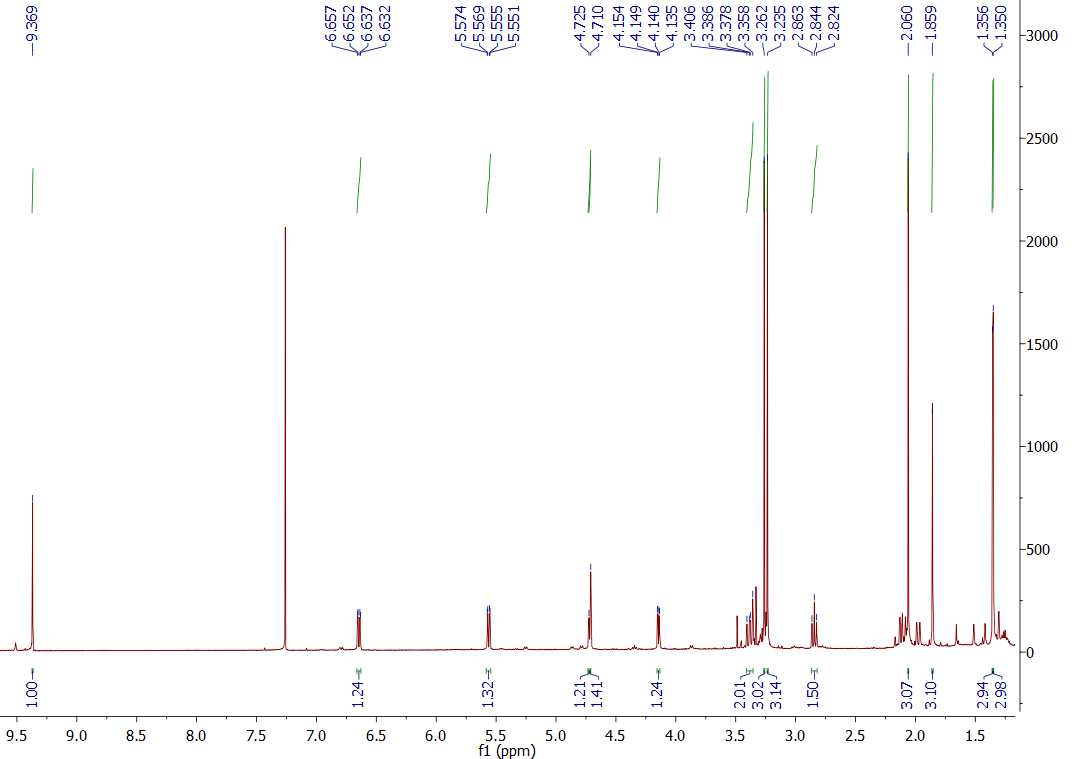


**Supplementary Figure 7.** ^1^H NMR spectrum of pestalothenin B (**2**; 600 MHz, CDCl_3_)


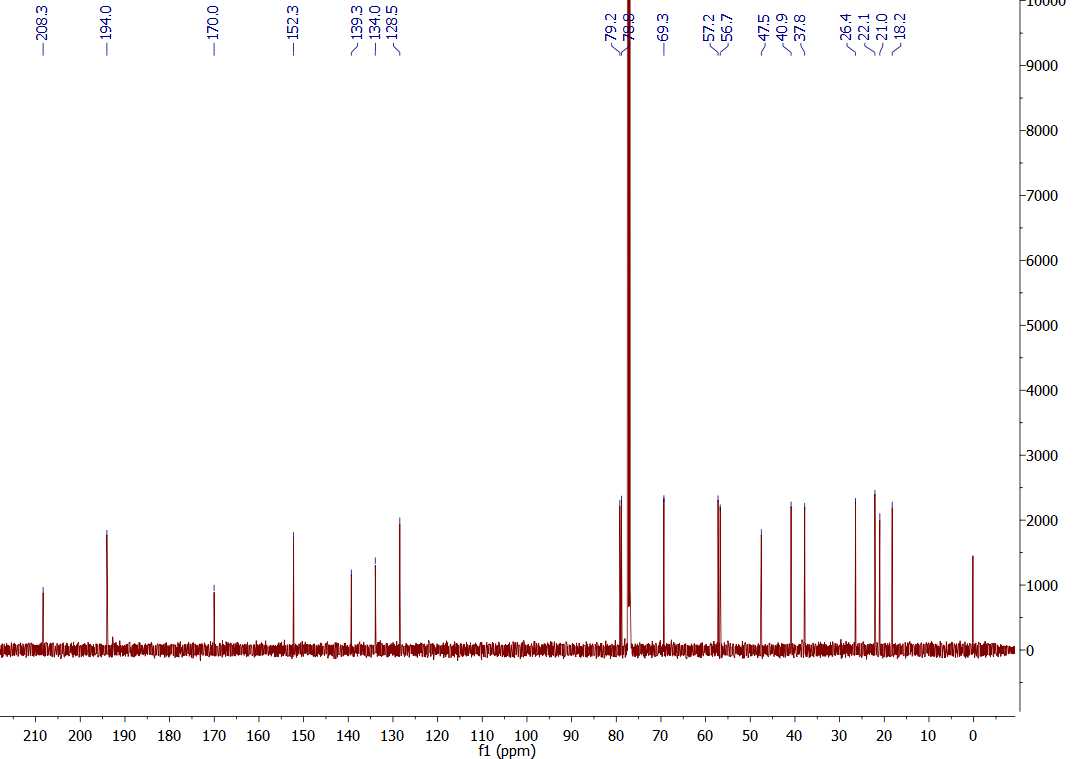


**Supplementary Figure 8.** ^13^C NMR spectrum of pestalothenin B (**2**; 150 MHz, CDCl_3_)


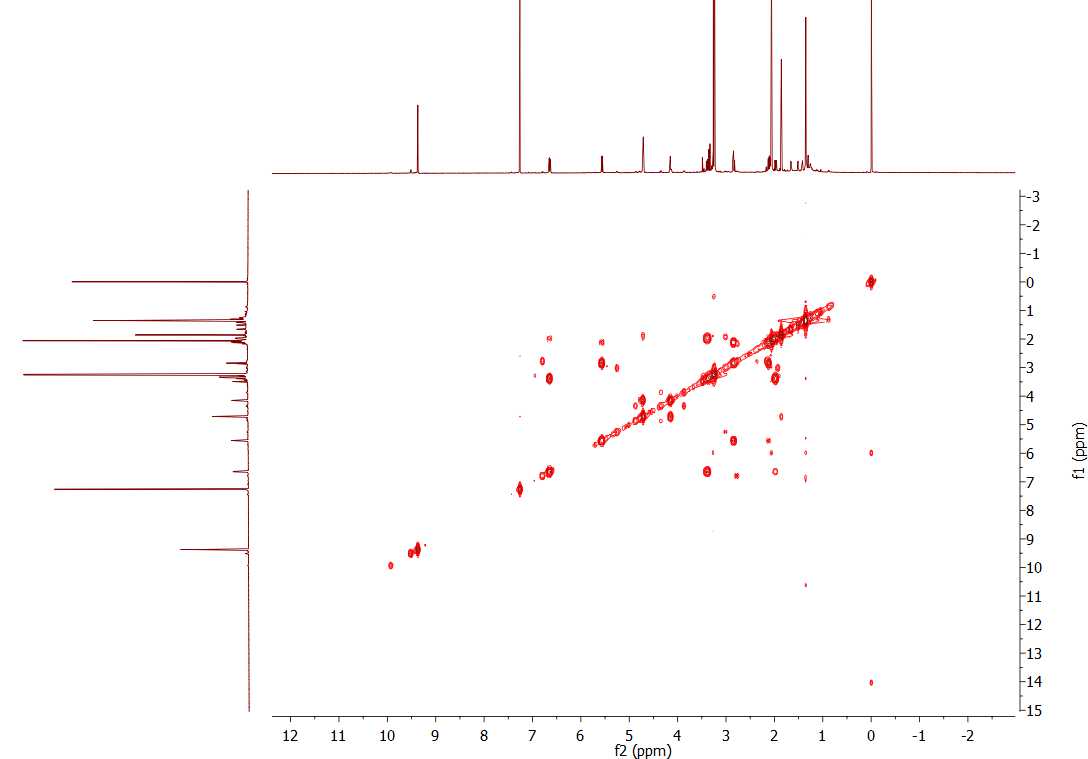


**Supplementary Figure 9.** ^1^H-^1^H COSY spectrum of pestalothenin B (**2**; 600 MHz, CDCl_3_)


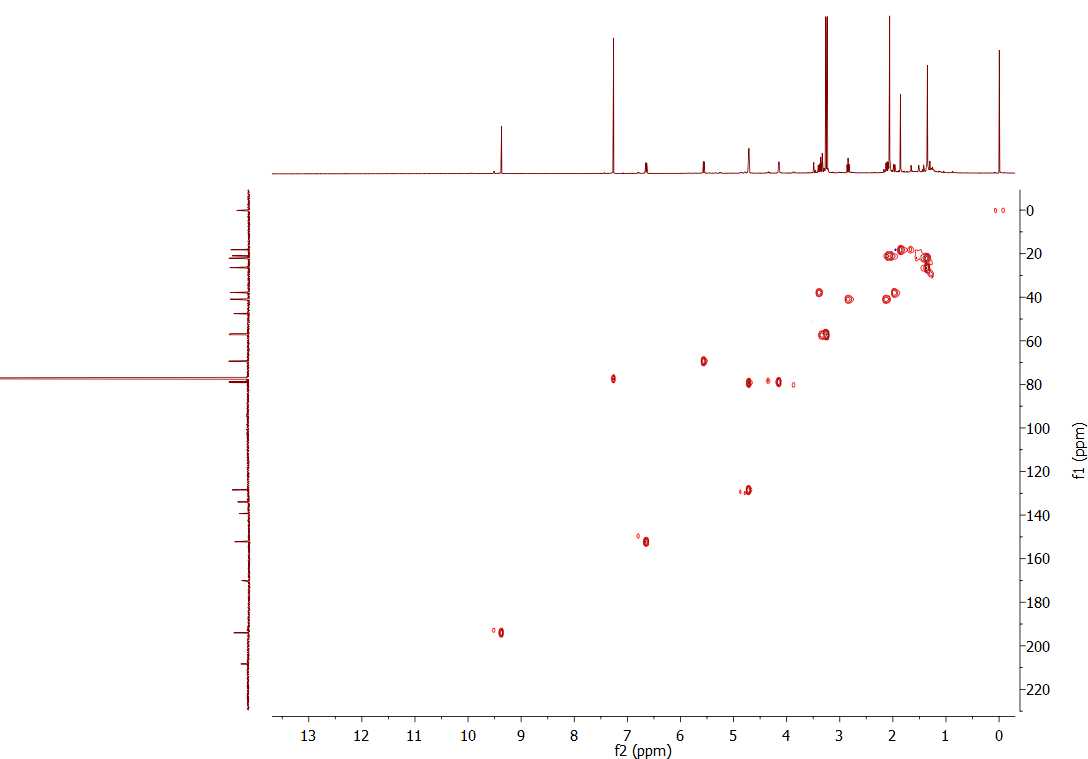


**Supplementary Figure 10.** HSQC spectrum of pestalothenin B (**2**; 600 MHz, CDCl_3_)


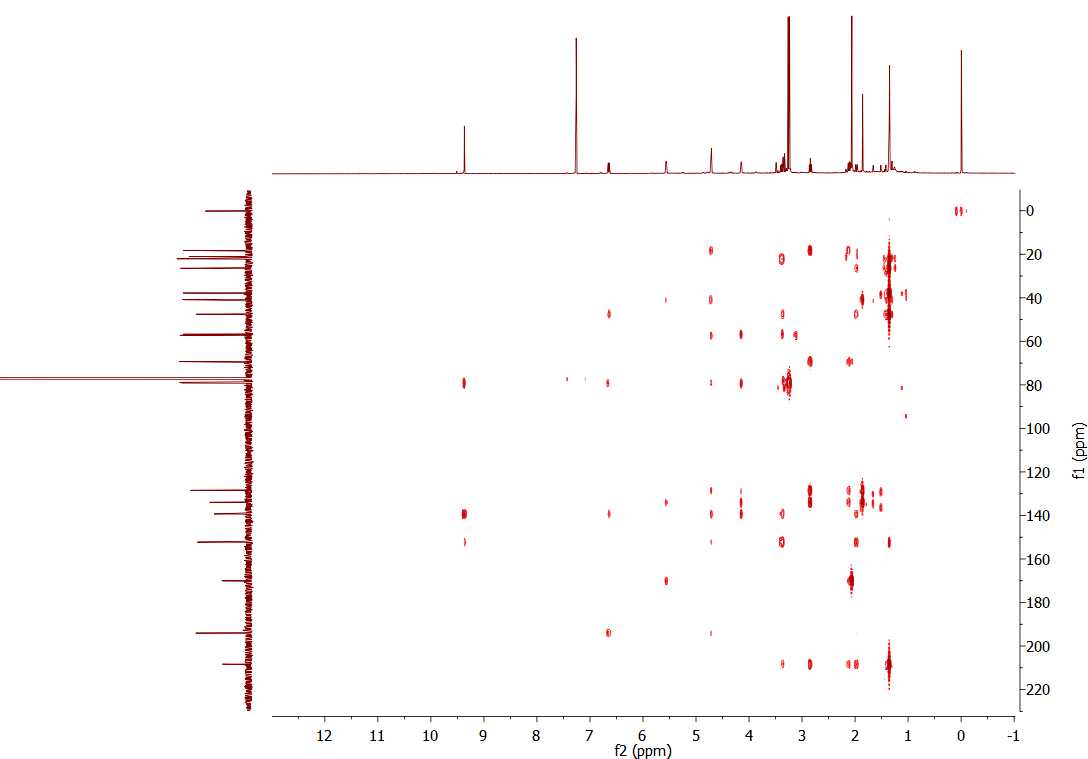


**Supplementary Figure 11.** HMBC spectrum of pestalothenin B (**2**; 600 MHz, CDCl_3_)


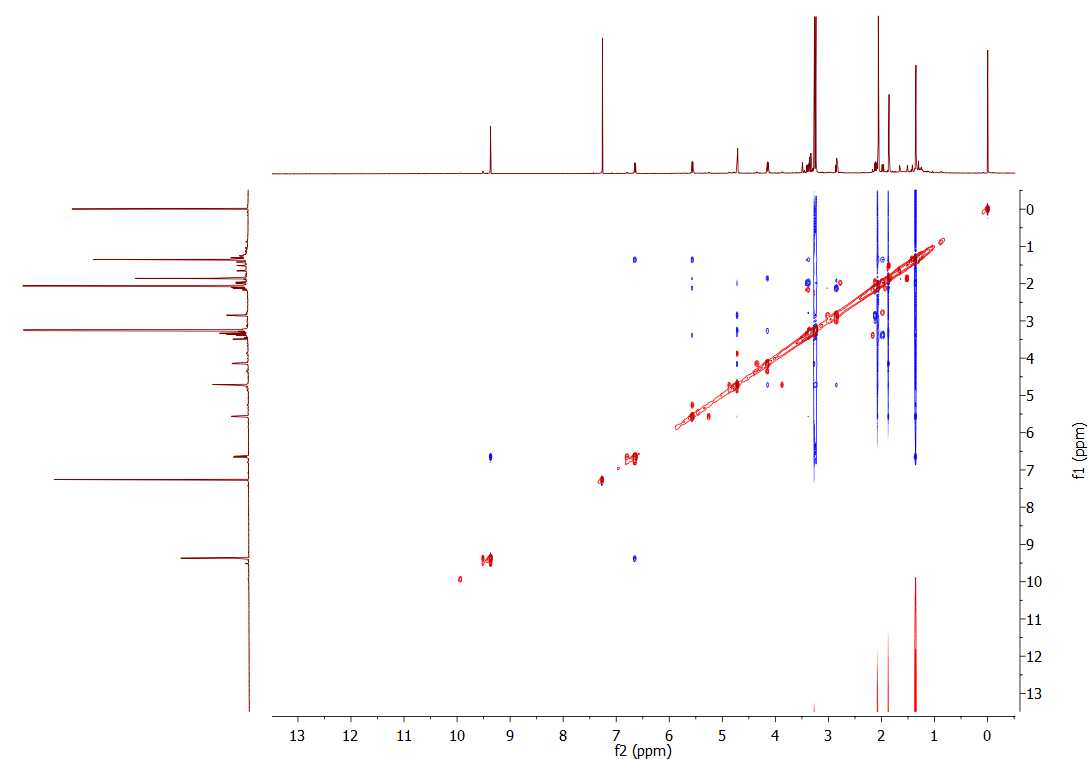


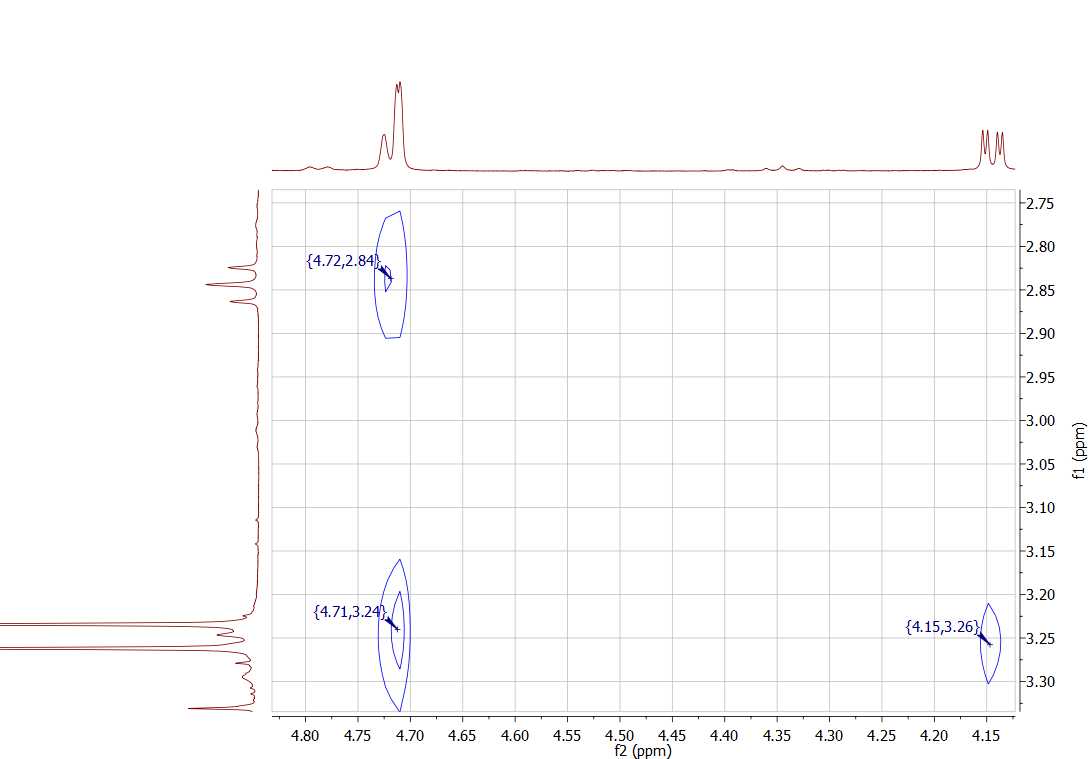


**Supplementary Figure 12.** NOESY spectrum of pestalothenin B (**2**; 600 MHz, CDCl_3_)


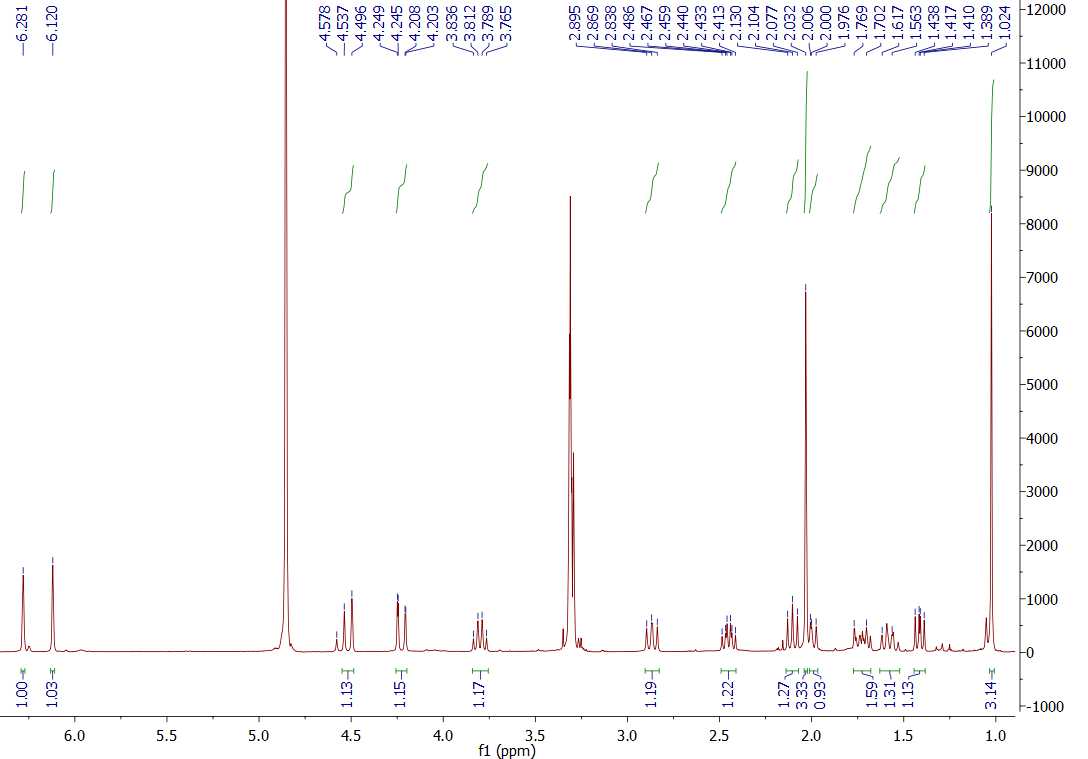


**Supplementary Figure 13.** ^1^H NMR spectrum of pestalothenin C (**3**; 400 MHz, methanol-*d*_4_)


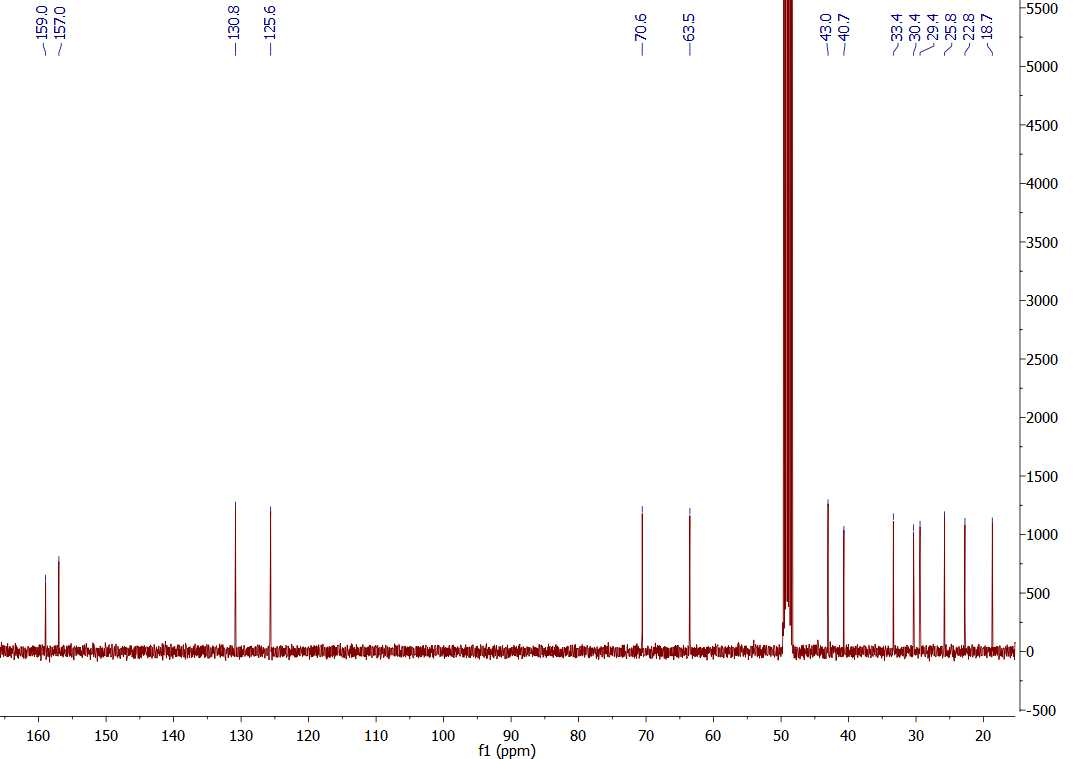


**Supplementary Figure 14.** ^13^C NMR spectrum of pestalothenin C (**3**; 100 MHz, methanol-*d*_4_)


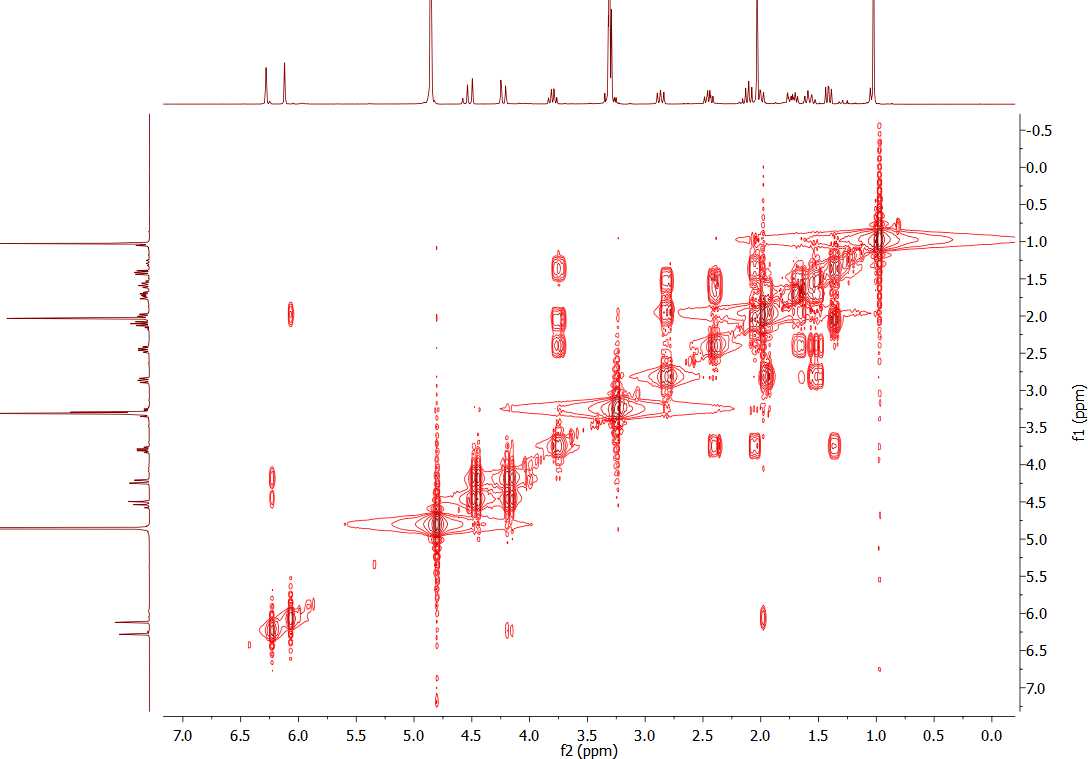


**Supplementary Figure 15.** ^1^H-^1^H COSY spectrum of pestalothenin C (**3**; 400 MHz, methanol-*d*_4_)


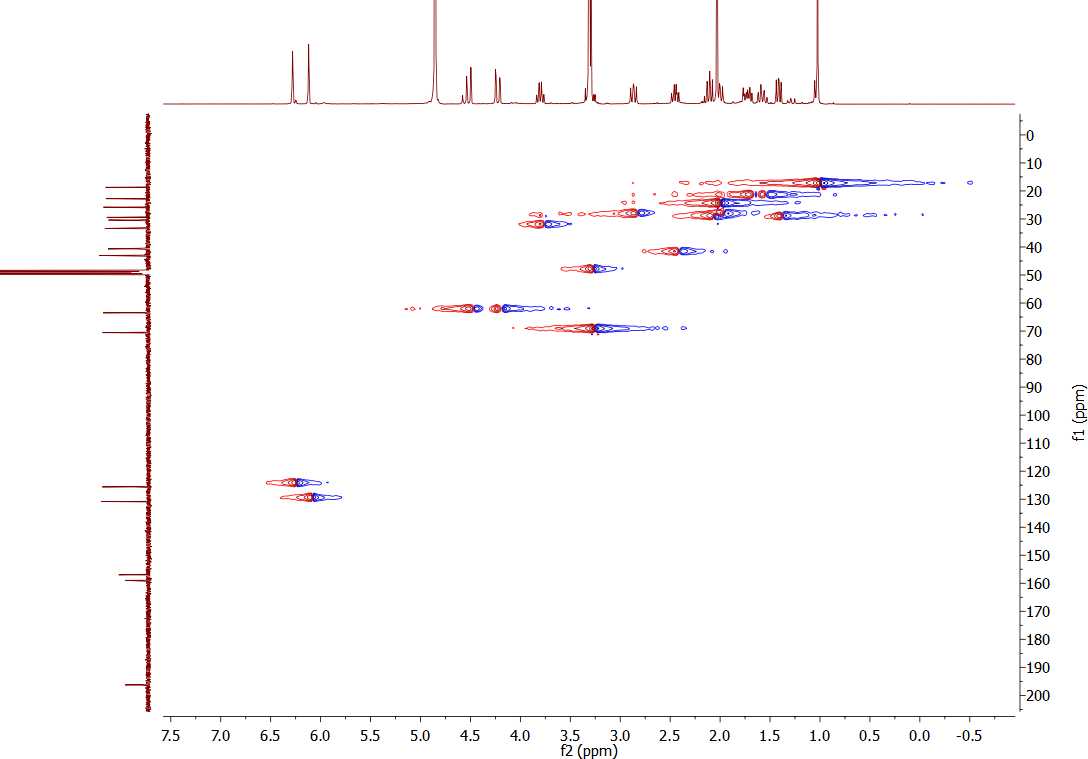


**Supplementary Figure 16.** HSQC spectrum of pestalothenin C (**3**; 400 MHz, methanol-*d*_4_)


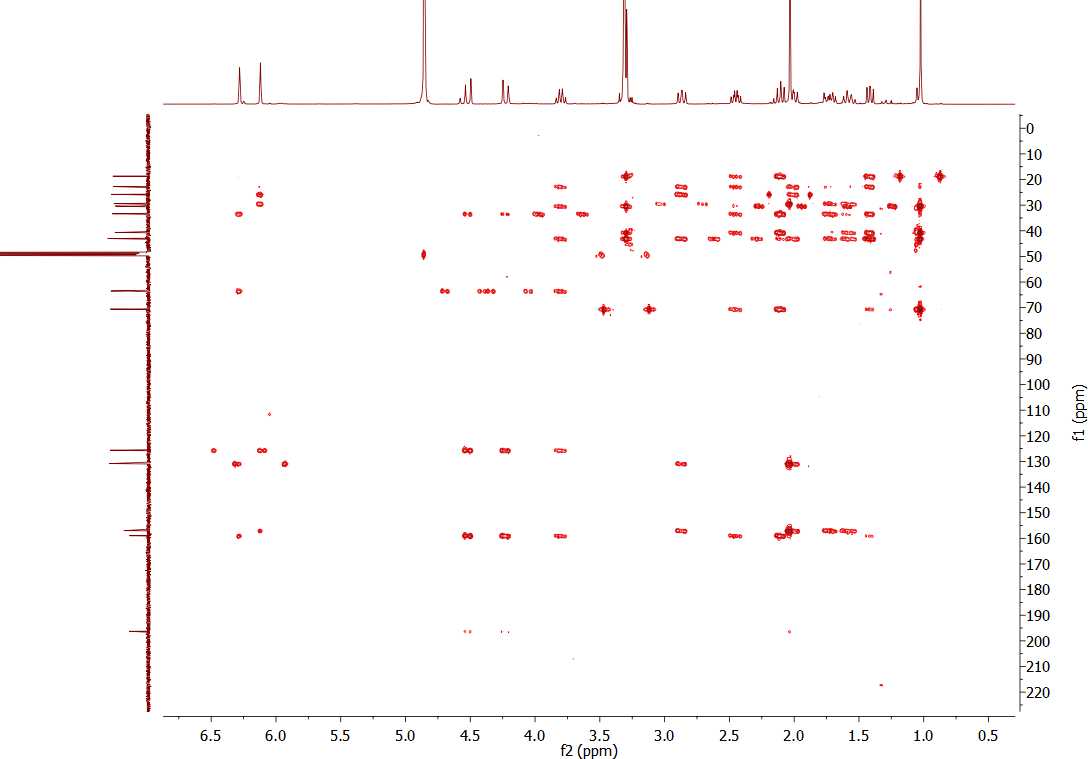


**Supplementary Figure 17.** HMBC spectrum of pestalothenin C (**3**; 400 MHz, methanol-*d*_4_)


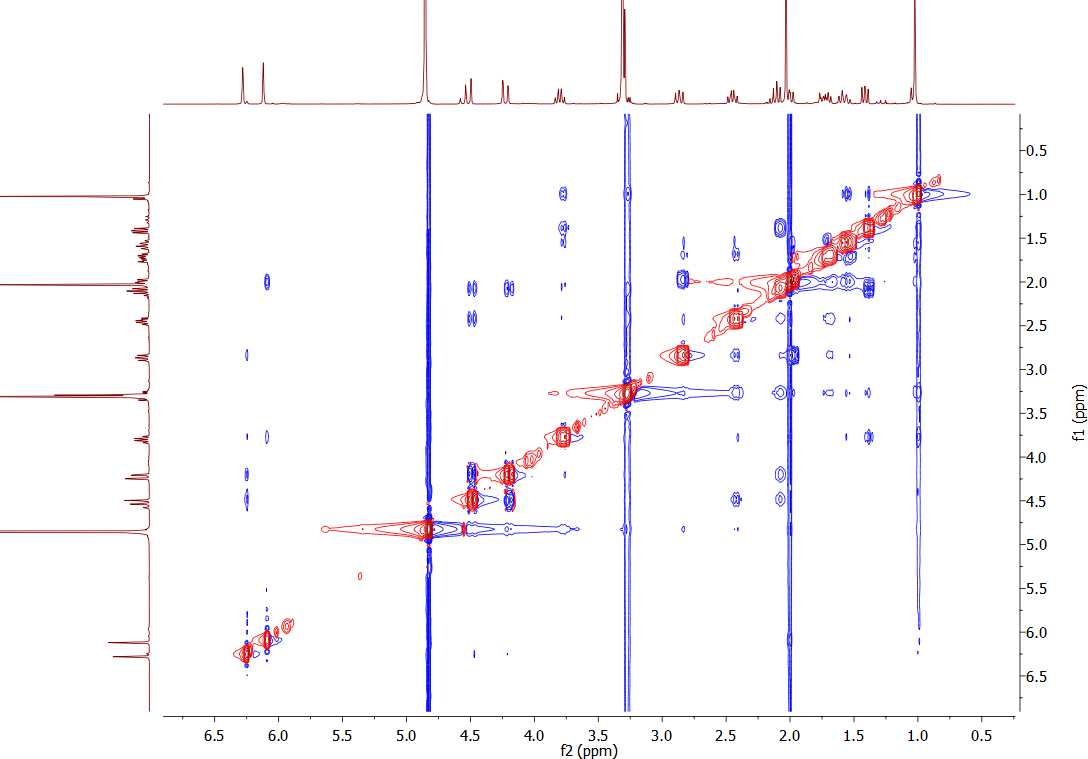


**Supplementary Figure 18.** NOESY spectrum of pestalothenin C (**3**; 400 MHz, methanol-*d*_4_)

|  | |  | |
| --- | --- | --- | --- |
| Conformers | Populations | Conformers | Populations |
| 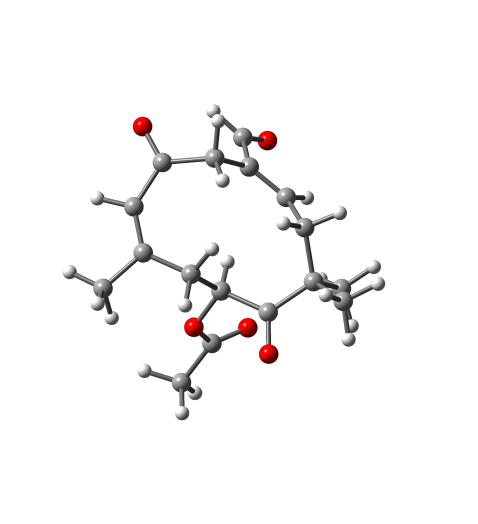 | 55% | 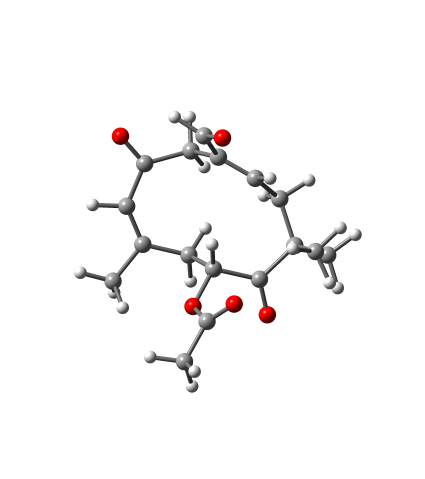 | 55% |
| 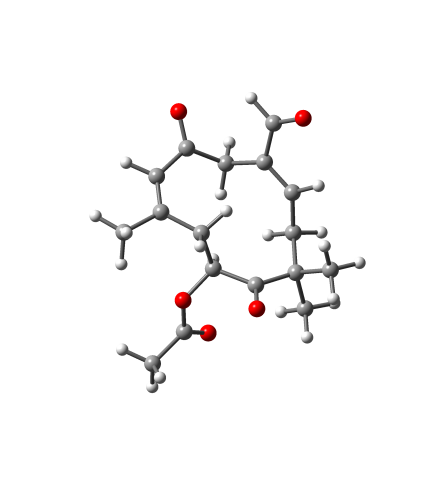 | 45% | 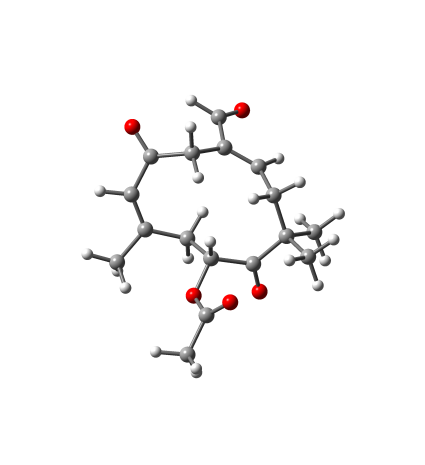 | 45% |

|  | |  | |
| --- | --- | --- | --- |
| Conformers | Populations | Conformers | Populations |
| 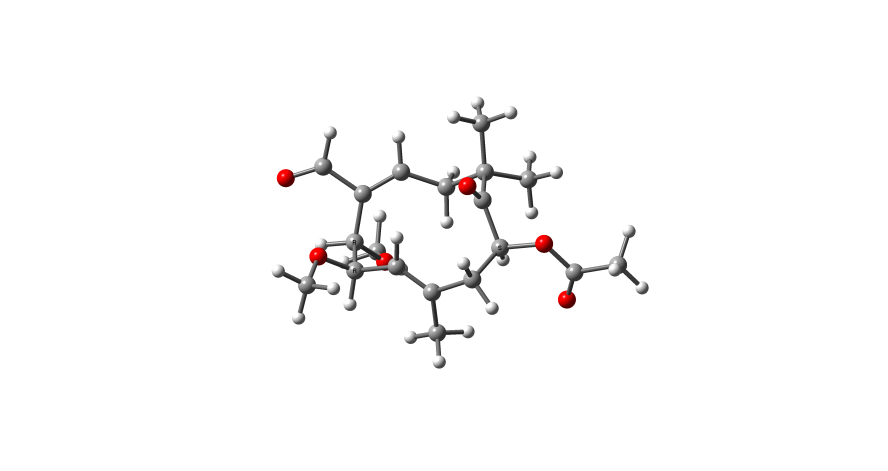 | 75% | 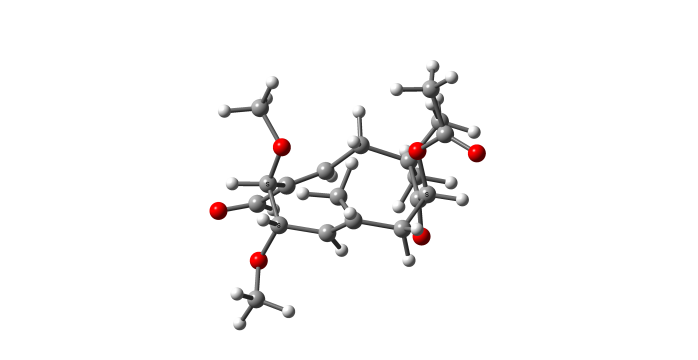 | 38% |
| 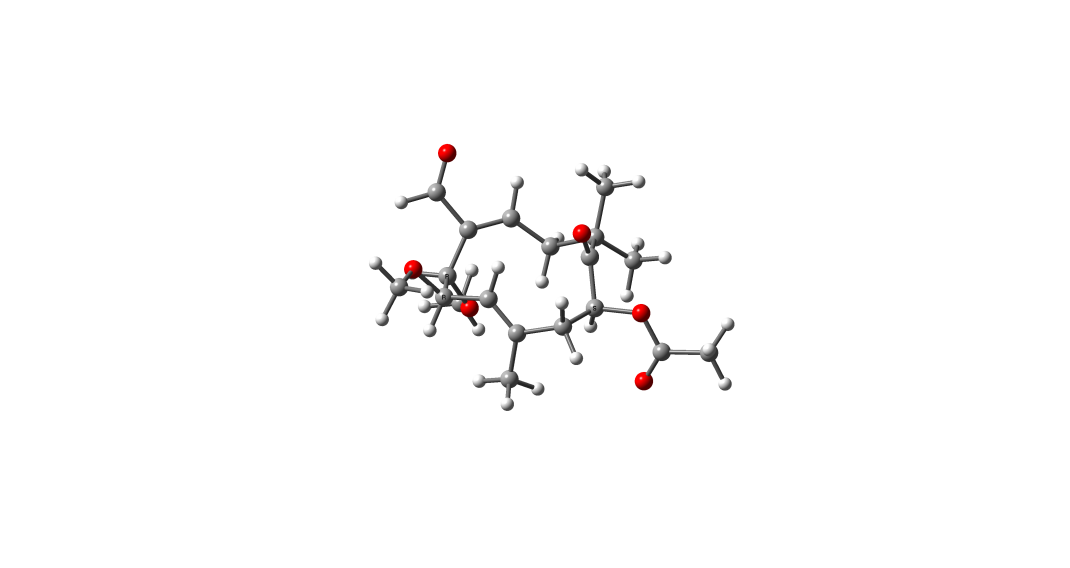 | 25% | 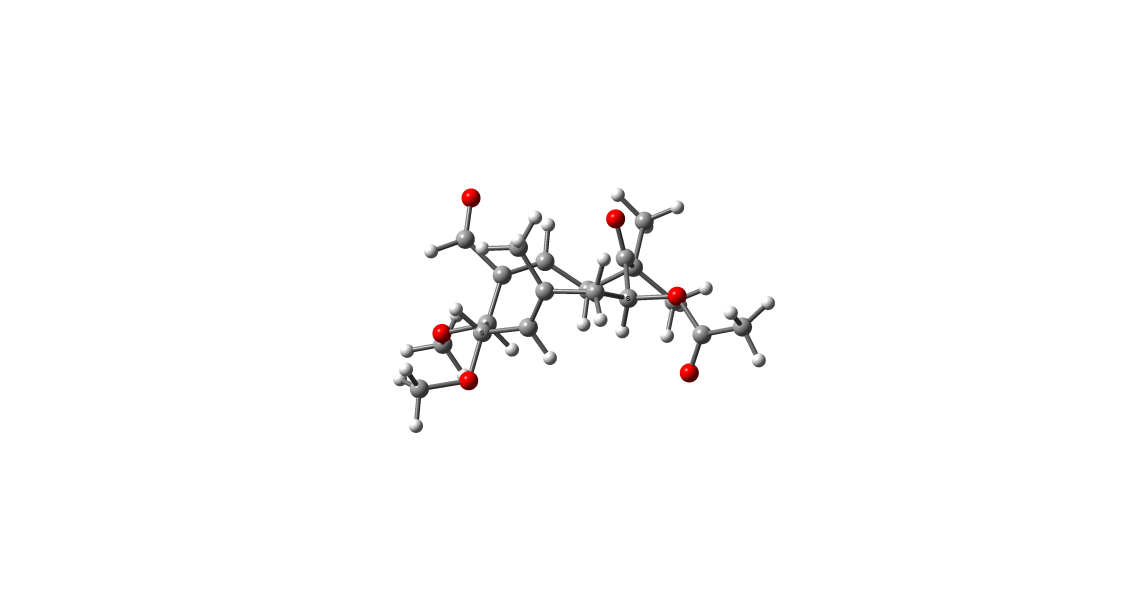 | 23% |
| 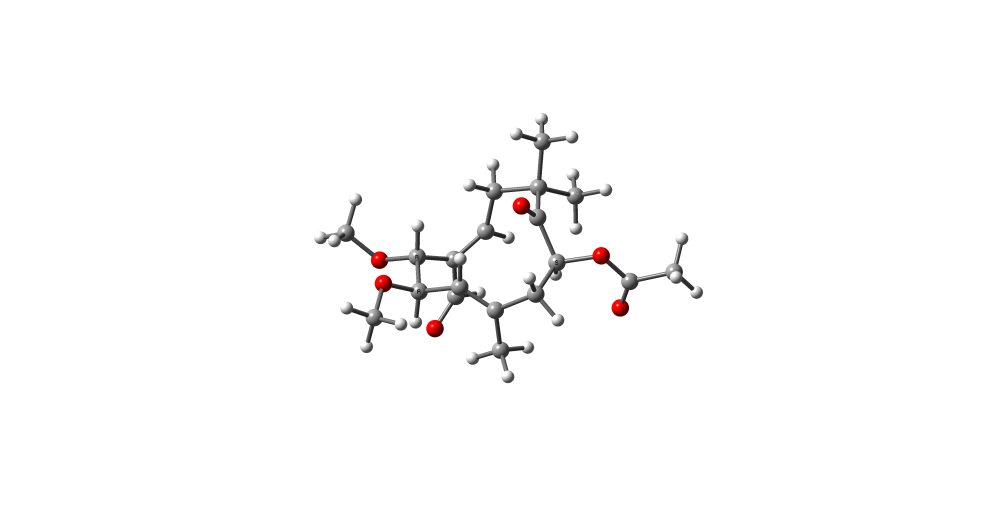 | 1% | 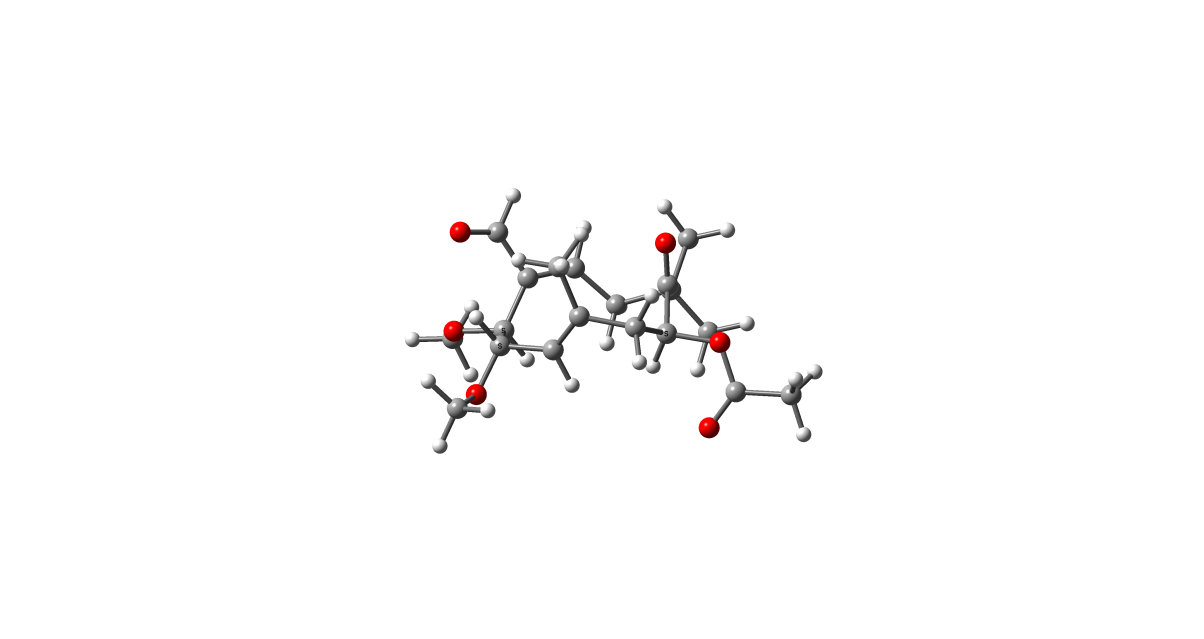 | 22% |
|  |  | 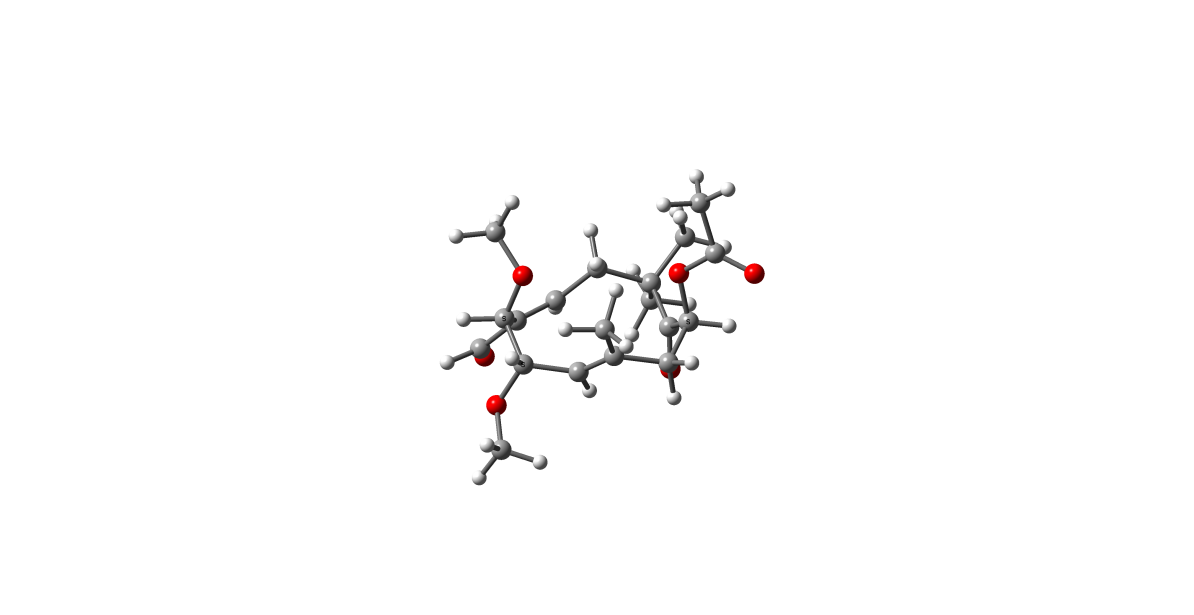 | 10% |
|  |  | 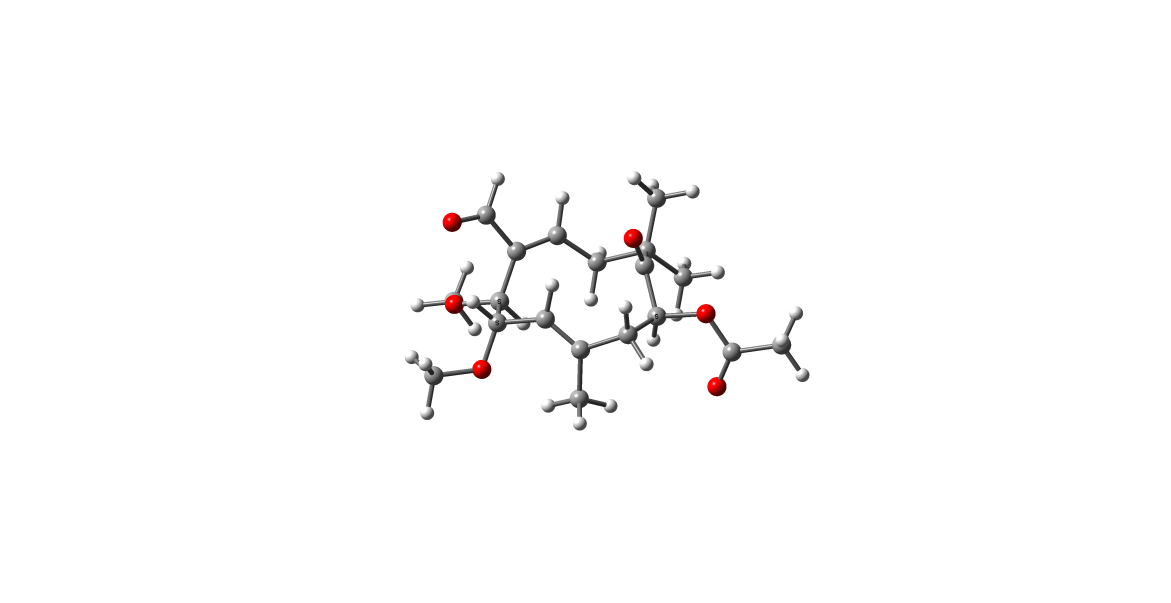 | 7% |
|  | | | |
| Conformers | | Populations | |
| 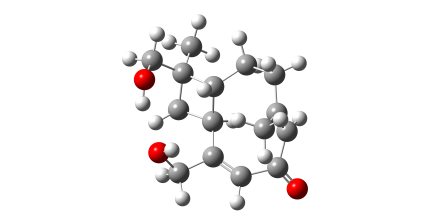 | | 49.67% | |
| 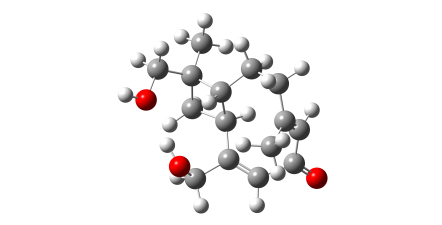 | | 50.33% | |

**Supplementary Figure 19.** ECD conformers of pestalothenins A–C (**1**–**3**)


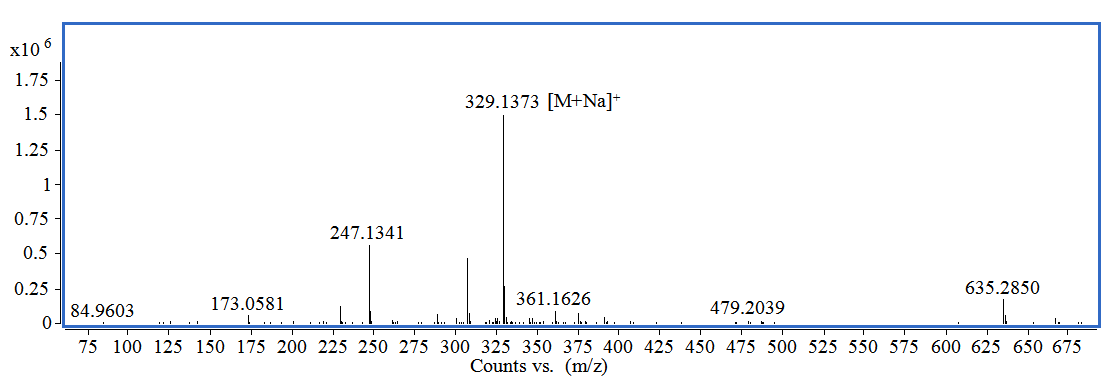


**Supplementary Figure 20.** HRESIMS spectrum of pestalothenin A (**1**)


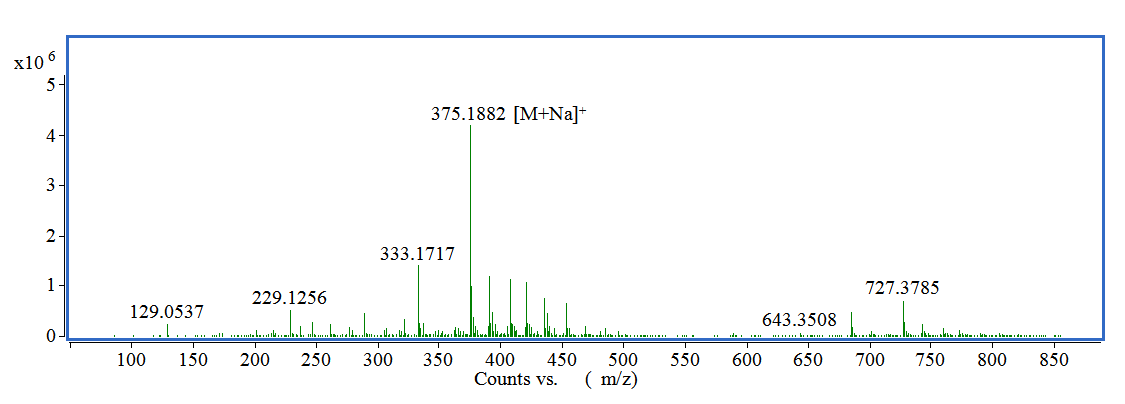


**Supplementary Figure 21.** HRESIMS spectrum of pestalothenin B (**2**)


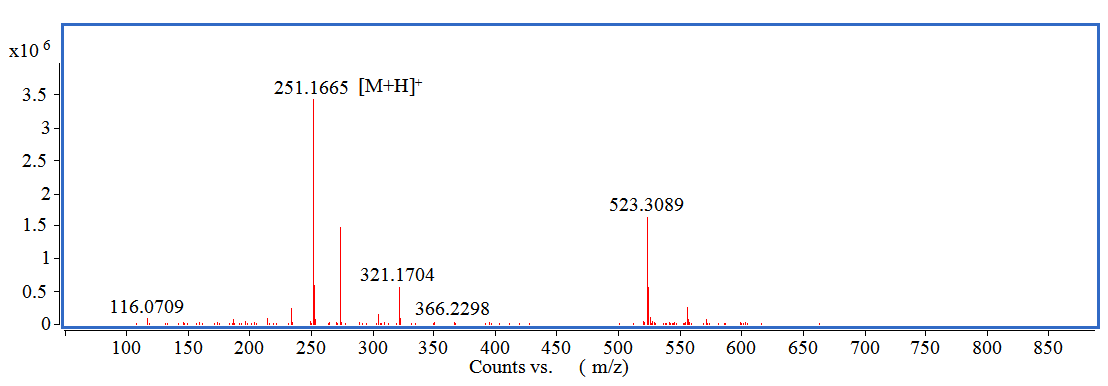


**Supplementary Figure 22.** HRESIMS spectrum of pestalothenin C (**3**)

**Supplementary Figure 23.** Experimental ECD spectrum of pestalothenin A (**1**) in MeOH

**Supplementary Figure 24.** Experimental ECD spectrum of pestalothenin B (**2**) in MeOH

**Supplementary Figure 25.** Experimental ECD spectrum of pestalothenin C (**3**) in MeOH
